# An Investigation for Large Volume, Focal Blood-Brain Barrier Disruption with High-Frequency Pulsed Electric Fields

**DOI:** 10.3390/ph14121333

**Published:** 2021-12-20

**Authors:** Melvin F. Lorenzo, Sabrina N. Campelo, Julio P. Arroyo, Kenneth N. Aycock, Jonathan Hinckley, Christopher B. Arena, John H. Rossmeisl, Rafael V. Davalos

**Affiliations:** 1 Department of Biomedical Engineering and Mechanics, Virginia Tech, Blacksburg, VA 24061, USA; scampelo@vt.edu (S.N.C.); jparroyo@vt.edu (J.P.A.); kna@vt.edu (K.N.A.); carena@vt.edu (C.B.A.); davalos@vt.edu (R.V.D.); 2 Department of Small Animal Clinical Sciences, Virginia-Maryland College of Veterinary Medicine, Blacksburg, VA 24061, USA; hinckley@vt.edu (J.H.); jrossmei@vt.edu (J.H.R.J.)

**Keywords:** blood-brain barrier disruption, electroporation, finite element methods, Gadolinium, Evans blue dye, T1-weighted MRI, treatment planning, pulsed field ablation, tissue ablation

## Abstract

The treatment of CNS disorders suffers from the inability to deliver large therapeutic agents to the brain parenchyma due to protection from the blood-brain barrier (BBB). Herein, we investigated high-frequency pulsed electric field (HF-PEF) therapy of various pulse widths and interphase delays for BBB disruption while selectively minimizing cell ablation. Eighteen male Fisher rats underwent craniectomy procedures and two blunt-tipped electrodes were advanced into the brain for pulsing. BBB disruption was verified with contrast T1W MRI and pathologically with Evans blue dye. High-frequency irreversible electroporation cell death of healthy rodent astrocytes was investigated in vitro using a collagen hydrogel tissue mimic. Numerical analysis was conducted to determine the electric fields in which BBB disruption and cell ablation occur. Differences between the BBB disruption and ablation thresholds for each waveform are as follows: 2-2-2 μ s (1028 V/cm), 5-2-5 μ s (721 V/cm), 10-1-10 μ s (547 V/cm), 2-5-2 μ s (1043 V/cm), and 5-5-5 μ s (751 V/cm). These data suggest that HF-PEFs can be fine-tuned to modulate the extent of cell death while maximizing peri-ablative BBB disruption. Furthermore, numerical modeling elucidated the diffuse field gradients of a single-needle grounding pad configuration to favor large-volume BBB disruption, while the monopolar probe configuration is more amenable to ablation and reversible electroporation effects.

## 1. Introduction

The blood-brain barrier (BBB) is primarily composed of tight junction proteins (occludin and claudins) and central nervous system (CNS) endothelial cells which form a protective barrier isolating systemic neurotoxins, macromolecules, and infectious particles from the brain parenchyma [1]. While this structure is critical for maintaining brain homeostasis, the BBB poses a challenge for intracranial drug delivery [2]. Passive diffusion across the BBB favors small lipid-soluble molecules, while large molecular weight solutes are transported via highly selective active transport mechanisms [3]. This selectivity often leads essential drug molecules designed to target malignant cells in the brain to be screened out, presenting an obstacle for the treatment of intracranial diseases including brain cancers, Alzheimer’s disease, Parkinson’s disease, and targeting drug-resistant epileptic foci [4,5,6]. For these diseases, the BBB can be locally compromised but typically does not cover the diseased area, making large, focal BBB disruption (BBBd) therapies a good candidate for treating these conditions [7,8,9].

Treatment options for enhancing the delivery of macromolecules into the brain parenchyma include convection-enhanced delivery (CED) and transcranial focused ultrasound (FUS) combined with microbubbles. CED seeks to bypass the BBB altogether [10,11]. This is accomplished by inserting a hollow catheter into the target tissue and using pressure-driven flow to deliver a variety of therapeutic agents from nanoparticles (<100 nm) [12] to monoclonal antibodies [13]. The PRECISE trial demonstrated the objective efficacy of CED for the delivery of cintredekin besudotox [14], though challenges facing CED are the potential for perfusate reflux, intricate catheter placement, and lengthy treatment sessions due to relatively slow infusion rates. Transcranial FUS combined with microbubbles offers versatile and non-invasive BBBd through the disassembly of tight junction proteins [15], however, BBB recovery typically occurs within a few hours after FUS [16], potentially limiting practical treatment windows. These drawbacks highlight the need for an alternative approach to enhanced drug delivery and expand the intracranial surgical armamentarium.

To enhance CNS drug delivery, previous studies support the use of electroporation-based therapies (EBTs) to focally [17] and transiently [18] disrupt the blood-brain barrier [19]. EBTs are a collection of therapies that employ pulsed electric fields (PEFs) to increase the transmembrane potential of a cell [20]. EBTs have been studied for their ability to enhance drug and gene delivery as well as ablate neoplastic and non-neoplastic tissues [21]. Notably, EBT tissue ablation in neoplastic tissue has been shown to induce systemic antitumor immunity [22,23]. Within the CNS, EBTs and PEF treatments are an emerging modality for transporting chemotherapeutics across the BBB [24,25] for the treatment of intracranial diseases. Specifically, high-frequency irreversible electroporation (H-FIRE) is an EBT capable of disrupting the BBB [26] and inducing selective cell death between malignant and healthy phenotypes [27]. The safety and feasibility of tissue ablation with H-FIRE have been investigated for the treatment of intracranial meningioma in a spontaneous canine brain tumor model [28], while the predecessor to this technology, named irreversible electroporation (IRE), has demonstrated the efficacious ablation of high-grade gliomas in canine patients presenting with spontaneous brain tumors [19].

Within the scope of tissue ablation, the transition from long monopolar pulses (first generation IRE) to the high-frequency bipolar pulses (used with H-FIRE) was motivated by a need to reduce skeletal muscle and nerve excitation during treatment [29,30,31]. Additionally, bipolar pulses reduce electrochemical effects including bubble production from electrolysis [32] and ionic corrosion of the electrode surface, which induce pH changes that modify the biochemical responses of electroporated cells [33,34]. Emphasis has been placed on investigating the effects of bipolar pulse waveform on cell electroporation, viability, and nerve excitation, though minimal work has been conducted to investigate the effects of HF-PEF pulsing parameters in the CNS. There is an interest to develop HF-PEFs to elicit large volumes of BBBd, while minimizing tissue ablation effects in applications where this may be desired. Specifically, large volume BBB disruption can be used for the clearance of protein aggregates which have been found to correlate with the incidence of Alzheimer’s disease, as well as inducing neuromodulation for diseases such as Parkinson’s and epilepsy.

Prior studies have demonstrated that HF-PEF waveforms with longer pulse widths induce larger regions of cell death, whereas a relatively short interphase delay may induce a “cancellation” effect on the bipolar pulses, thereby reducing cell ablation [35]. Building upon prior BBBd studies [18,26,36] and in the interest of adapting HF-PEFs therapy for clinically relevant (>3 cm) BBB disruption in the absence of ablation, this study investigates the effects of electrode configuration, HF-PEF pulse width, interphase delay, and implicit frequency content on BBB disruption, cell ablation, cell reversible electroporation, and nerve excitation. In this study, high-frequency pulsed electric fields were utilized for inducing three distinct physiological phenomena: (1) cell ablation by means of high-frequency irreversible electroporation; (2) cell reversible electropermeabilization by means of electroporation; and (3) blood-brain barrier disruption presumably through the disruption of tight junction proteins by means of HF-PEFs. The nomenclature to define the burst of biphasic PEFs is: positive phase–interphase delay–negative phase (units defined in μ s) (see Figure S1). Here, the pulse width and the interphase delay were varied to modulate the extent of cell death in relation to BBB disruption.

## 2. Results

### 2.1. Study Overview

Two experimental data sets were collected: (1) in vivo characterization of BBBd following HF-PEFs treatment with various pulse widths and delays and (2) in vitro characterization of ablation and reversible electroporation effects in healthy rodent astrocytes. A third in silico data set was collected to quantify nerve excitation following HF-PEFs treatment with various pulse widths and delays; these data were simulated using a spatially extended nonlinear node (SENN) [29,37]. As ablation, reversible electroporation, BBB disruption, and nerve excitation are induced by external HF-PEFs, it is possible to numerically quantify the threshold electric fields which elicit these effects. After quantifying these threshold fields, a numerical analysis was conducted to investigate these effects in clinically relevant scenarios and to determine a pulsing scheme to maximize focal blood-brain barrier disruption with minimal ablation and nerve excitation.

### 2.2. Quantifying HF-PEF-Mediated BBB Disruption in Healthy Rodent Brain In Vivo

**Figure 1:**
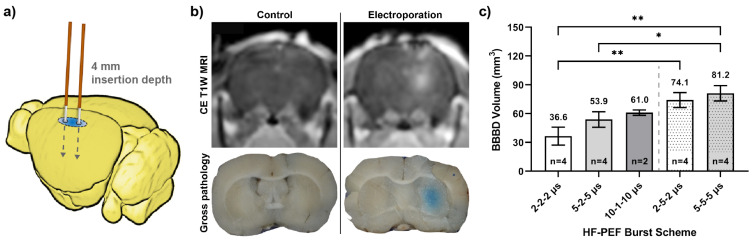
Summary of HF-PEF BBB disruption, resulting in significant diffusion of normally impermeant Gd-EBD; (**a**) schematic depicting electrode insertion trajectory in rodent brain tissue; (**b**) coronal view of BBB disruption as seen on a plane traversing the electrode insertion track and as shown by accumulation of Gd-EBD with contrast enhanced T1W MRI and gross tissue sections (sham vs. representative 5-5-5 μ s treatment). Without HF-PEFs, no uptake of Gd-EBD is seen; (**c**) volumetric measurements determined from gross tissue sections, * *p* ≤ 0.05 and ** *p* ≤ 0.01.

Male Fischer rats underwent aseptic surgery and two needle-electrodes were used to deliver the electric pulses (Figure 1a); this configuration produces an electric field that radiates outward from the energized metal surface. Rodents were subsequently sacrificed and imaged at a time point 1 h following treatment. The permeability of the BBB was assessed using contrast-enhanced (CE) T1W MRI (gadopentetate dimeglumine, Gd), pathologically using gross tissue sections (Evans blue dye, EBD), and in select rats by measuring the EBD fluorescence in both the serum and brain parenchyma.

Treatment was administered with waveforms summarized in Table 1. To highlight the effects of HF-PEF waveforms on BBBd, all treatment groups received the same electrical dosage with 240 V applied, 200 bursts administered, 100 μ s on-time per burst, and bursts delivered at a rate of 1 burst per second. One metric for BBBd was the measurement of EBD fluorescence, both in the serum (processed from blood samples taken immediately following euthanasia) and in the brain parenchyma (“cerebral”, Table 1). In all cases, the serum EBD fluorescence was greater than 1300 μ g/g, indicating high systemic EBD for all pulse protocols. The intraparenchymal EBD was 0.20 μ g/g in the sham (n = 1) and greater than 14.1 μ g/g in all other cases. In the 2-2-2 μ s (n = 2), 5-2-5 μ s (n = 2), 2-5-2 μ s (n = 2), and 5-5-5 μ s (n = 2), the intracranial EBD was 14.1 ± 0.2 μ g/g, 15.2 ± 0.1 μ g/g, 16.9 ± 0.1 μ g/g, and 18.5 ± 0.3 μ g/g, respectively. These results indicate HF-PEFs are required for a significant diffusion of EBD from systemic circulation in the brain (Figure 1b), though these measurements do not provide spatial information of the BBBd relative to the applied electric field.

BBBd volumes of EBD were reconstructed from digital photomicrographs of coronal brain sections, while volumes of Gd contrast enhancement were reconstructed from MRI scans (Table 1). Sample size numbers for these BBBd measurements are as follows (EBD, Gd): 2-2-2 μ s (n = 4, n = 2), 5-2-5 μ s (n = 4, n = 2), 10-1-10 μ s (n = 2, n = 2), 2-5-2 μ s (n = 4, n = 2), and 5-5-5 μ s (n = 4, n = 2). A Wilcoxon matched-pairs signed-rank test was used to compare volumetrics from MRI and gross pathology methodologies by pairing the applied waveforms. The test indicates that the volumetrics from either method were not significantly different from one another (*p* = 0.313). A Kruskal–Wallace (KW) test with an uncorrected Dunn’s multiple comparisons test was used to highlight the differences among all pathological BBBd groups, regardless of pulse width and interphase delay, as shown in Figure 1c. A two-way ANOVA with Bonferroni’s multiple comparisons test was used to test differences between pulse widths (*p* = 0.0206) and interphase delays (*p* < 0.0001). Interestingly, Bonferroni’s multiple comparison indicates differences when comparing 2 μ s and 5 μ s interphase delay groups, though when comparing the effects of pulse width (2-2-2 μ s vs. 5-2-5 μ s and 2-5-2 μ s vs. 5-5-5 μ s), no differences were detected (*p* = 0.1239 and *p* = 0.1239, respectively). This analysis indicates that BBBd is strongly correlated with interphase delay, whereas pulse width only moderately affects BBBd.

Volumes were subsequently mapped to the numerical electric field distribution to quantify the BBB disruption electric field threshold (EFT), the minimum electric field required to elicit BBB disruption with HF-PEFs (see Section 2.4).

### 2.3. Characterization of Healthy Rodent Astrocyte Cell Death and Reversible Electroporation In Vitro

**Figure 2:**
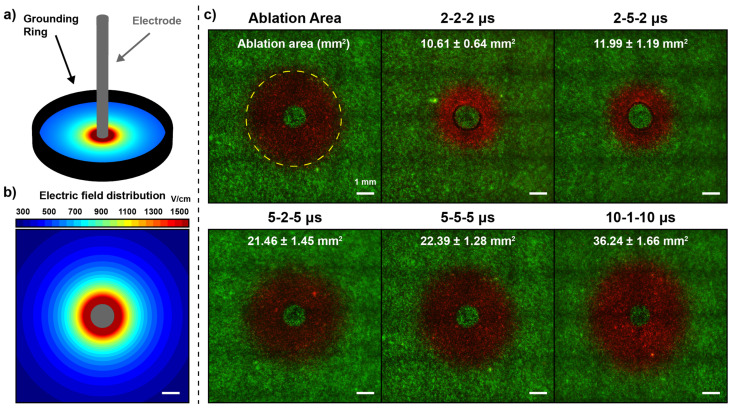
A collagen hydrogel scaffold was leveraged to determine the electric field threshold of cell death for healthy rodent astrocytes: (**a**) a single-needle grounding ring electrode configuration induces a (**b**) rotationally symmetric electric field distribution; (**c**) ablation is quantified as the area of propidium iodide uptake for the 2-2-2 μ s, 2-5-2 μ s, 5-2-5 μ s, 5-5-5 μ s, and 10-1-10 μ s groups. The ablation area is mapped to a corresponding electric field and this field is the lethal electric field threshold. In all cases, 600 V was applied.

To complement the in vivo BBB disruption data set, a collagen hydrogel tissue mimic was utilized to determine the minimum electric fields necessary to elicit H-FIRE cell death in healthy rodent astrocytes (DI TNC1) in vitro. Cell death with H-FIRE is known as a transient process, with a full lesion developing in ~24 h [38]. As such, hydrogels seeded with DI TNC1 cells were maintained at a physiologic temperature (37 °C), pulsed, then incubated until staining for imaging at a 24 h time point. Pulsed electric fields were applied using a single-needle grounding ring electrode configuration, which produces a rotationally symmetric electric field distribution as seen in Figure 2a,b. Cell death was quantified as the circular area of propidium iodide uptake, a membrane impermeable dye. At 24 h, reversible electroporation effects have ceased, thus leaving only the cells with compromised membranes to uptake this dye (Figure 2c).

In parallel to these studies, the reversible electroporation of the cells was also quantified. In contrast to the ablation measurements, hydrogels were stained prior to pulsing, and electroporation areas were measured 45 min following treatment delivery as indicated by the time-lapse study (Figure S2). In both ablation and reversible electroporation groups, an equal energy HF-PEF pulsing protocol was administered (600 V, 100 μ s energized time, 100 bursts), therefore all in vitro data are directly comparable to one another (Figure S3).

Similar to the in vivo BBBd studies, the independent variables studied were the pulse widths and interphase delays. A one-way ANOVA with a Bonferroni’s multiple comparison was used to test for significant differences between all groups, regardless of pulse width and interphase delay, and is reported in the supplemental material (Figure S3). Visually, Figure 2c shows an enhanced ablation area in response to increased pulse width. Relative to the ablation area for the 2-2-2 μ s waveform, the ablation areas are increased by a factor of 2.02× for the 5-2-5 μ s waveform, 3.41× for the 10-1-10 μ s waveform, 1.13× for the 2-5-2 μ s waveform, and 2.11× for the 5-5-5 μ s waveform. A two-way ANOVA with Bonferroni’s multiple comparisons test was used to test differences between grouping similar pulse widths (*p* < 0.0001) and grouping similar interphase delays (*p* = 0.0085). Bonferroni’s multiple comparison indicates differences when comparing 2 μ s and 5 μ s pulse widths groups, though when comparing the effects of interphase delays (2-2-2 μ s vs. 2-5-2 μ s and 5-2-5 μ s vs. 5-5-5 μ s), no differences were detected (*p* = 0.4200 and *p* > 0.9999, respectively). This analysis indicates that the ablation area is strongly correlated with pulse width, whereas the interphase delay only moderately affects the ablation area.

In regard to reversible electroporation, relative to the electroporation area for the 2-2-2 μ s waveform, the electroporation areas are increased by a factor of 2.05× for the 5-2-5, 2.74× for the 10-1-10 μ s waveform, 1.06× for the 2-5-2 μ s waveform, and 1.97× for the 5-5-5 μ s waveform. These areas follow the same trends as those noted for ablation protocols (see supplemental Figure S3); however, larger reversible electroporation areas reported as lower electric fields are required to induce these effects [38]. A two-way ANOVA indicates statistical significance when pairing data by pulse width (*p* < 0.0001) but not when paring by interphase delay (*p* = 0.9166). This analysis indicates that reversible electroporation is strongly correlated with pulse width, whereas interphase delay does not significantly affect the reversible electroporation area.

These H-FIRE cell death and reversible electroporation area measurements were subsequently mapped to the numerical electric field distribution to quantify the ablation and reversible electroporation EFTs (see Section 2.4).

### 2.4. Electric Field Thresholds for BBB Disruption, Electroporation, Cell Death, and Nerve Excitation

**Figure 3:**
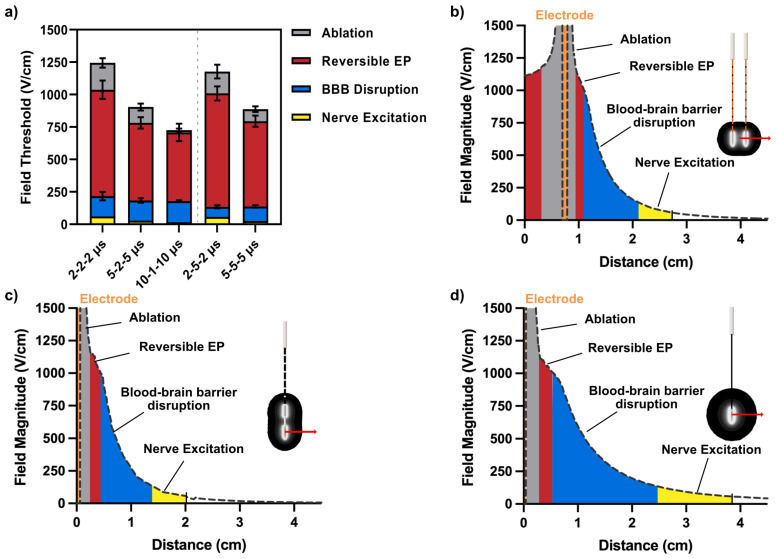
Delineation of biological phenomena elicited from PEF therapy for data collected in vivo and in vitro: (**a**) analysis of the electric field thresholds for cell ablation, reversible electroporation, BBB disruption, and nerve excitation demonstrates high delineation with effects further amplified by modulating the pulse width and interphase delay. (**b**–**d**) For the 2-5-2 μ s waveform, (**b**) the monopolar probe configuration capitalizes on high electric fields near and adjacent to the electrodes to induce large ablation and reversible electroporation, whereas (**d**) the single-needle grounding pad configuration capitalizes on a diffuse field gradient to reduce ablation and reversible electroporation effects and maximize BBB disruption. (**c**) While the bipolar probe has the advantages of a single insertion device, both ablation and BBB disruption areas are smaller and stay confined to the immediate electrode area.

Following the collection of BBBd volumetrics, ablation areas, and reversible electroporation areas, numerical methods were implemented to recapitulate the respective experimental setup and map the data to their corresponding electric field thresholds. The numerical models accounted for changes in conductivity due to electroporation effects, temperature changes, and frequency-dependent electrical conductivity. This resulted in a collection of EFTs for each of the respective HF-PEF phenomena as summarized in Table 2. In addition, a SENN nerve fiber model was implemented to estimate nerve excitation thresholds for the 2-2-2 μ s (64.64 V/cm), 5-2-5 μ s (28.56 V/cm), 10-1-10 μ s (16.0 V/cm), 2-5-2 μ s (57.0 V/cm), and 5-5-5 μ s (25.4 V/cm). A summary of effects organized by HF-PEF waveform is shown in Figure 3a.

Delineation between cell ablation and BBBd thresholds is quantified as the difference Δ E = E_Ablation_− E_BBBd_. The results of this difference for each waveform are as follows: 2-2-2 μ s ( Δ E = 1028.3 V/cm), 5-2-5 μ s ( Δ E = 721.2 V/cm), 10-1-10 μ s ( Δ E = 547.1 V/cm), 2-5-2 μ s ( Δ E = 1043.6 V/cm), and 5-5-5 μ s ( Δ E = 751.1 V/cm). This analysis implies that a greater delineation between BBBd and ablation can be achieved when implementing HF-PEFs of shorter pulse width.

A one-way ANOVA was used to compare all groups and a Bonferroni multiple comparisons to compare individual groups. This analysis is plotted in the supplemental material and was conducted for the cell ablation data set (Figure S3a,b), the reversible electroporation data set (Figure S3c,d), and the BBBd data set (Figure S4a,b). Secondly, a two-way ANOVA with Bonferroni’s multiple comparisons test was used to test differences of the electric field thresholds (separately analyzed for ablation, reversible electroporation, and BBB disruption) between grouping similar pulse widths (2 μ s and 5 μ s only), and grouping similar interphase delays (2 μ s and 5 μ s only).

#### 2.4.1. Pulse Width and Interphase Delay Influence the Ablation Electric Field Thresholds

The two-way ANOVA indicates statistical significance when pairing data by either pulse width (*p* < 0.0001) or interphase delay (*p* = 0.0040). Bonferroni’s multiple comparison indicates differences when comparing 2 μ s and 5 μ s pulse width groups (in all cases *p* < 0.0001). When comparing the effects of interphase delays (2-2-2 μ s vs. 2-5-2 μ s and 5-2-5 μ s vs. 5-5-5 μ s), lower level differences were detected (*p* = 0.0242 and *p* = 0.0242, respectively). This analysis indicates that the ablation EFT is strongly correlated with pulse width, whereas interphase delay still shows only a weak statistical significance.

#### 2.4.2. Pulse Width Has a Large Influence on the Reversible Electroporation Field Thresholds

The two-way ANOVA indicates statistical significance when pairing data by pulse width (*p* < 0.0001) but not by the interphase delay (*p* = 0.6698). Bonferroni’s multiple comparison indicates differences when comparing 2 μ s and 5 μ s pulse width groups (in all cases *p* < 0.0001). This analysis indicates the reversible electroporation EFT is strongly correlated with pulse width, whereas interphase delay does not significantly affect the reversible electroporation EFT.

#### 2.4.3. Interphase Delay Has a High Influence on the BBB Disruption Field Thresholds

The two-way ANOVA does not indicate statistical significance when pairing data by pulse width (*p* = 0.1969), however, it does indicate a difference when grouping by interphase delay (*p* = 0.0001). Bonferroni’s multiple comparison further indicates differences when comparing interphase delays: 2-2-2 μ s vs. 2-5-2 μ s with *p* = 0.0008 and 5-2-5 μ s vs. 5-5-5 μ s with *p* = 0.0008. This analysis indicates the BBBd EFT is strongly correlated with interphase delay, whereas pulse width does not significantly affect the BBBd EFT.

### 2.5. HF-PEF Waveform Modulates Extent of Cell Death While Maintaining Large BBB Disruption

**Figure 4:**
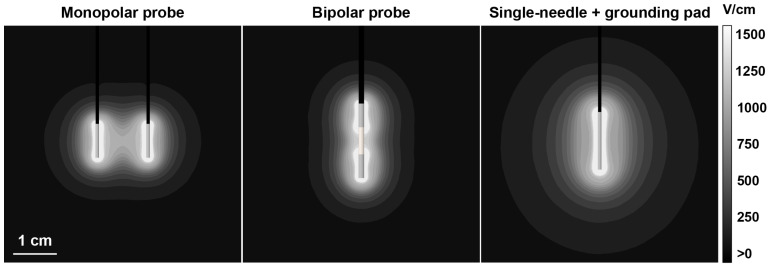
Representative electric field distributions for the dual monopolar probe, single insertion bipolar probe, and the single-needle distant grounding pad configuration. The electric field distribution is that of a 2-5-2 μ s waveform with 2 kV applied, simulated to include electroporation effects and coupled Joule heating effects.

To better understand the contributions of varying waveforms on electroporation effects and BBBd, a 3D finite element model was developed in COMSOL Multiphysics to simulate BBB disruption, ablation, reversible electroporation, and nerve excitation in three distinct electrode configurations. This included the dual monopolar probe configuration, the single insertion bipolar probe configuration, and the single-needle + distant grounding pad configuration (Figure 4).

The effects of HF-PEF waveforms were modeled in two ways: (1) the electrical conductivity sigmoid was specific for each waveform (Table 3), and (2) the electric field threshold for BBBd, ablation, reversible electroporation, and nerve excitation were specific for each waveform and are those determined in Section 2.4 (Table 2).

#### 2.5.1. Differences in Field Gradients between Electrode Types Can Be Exploited to Modulate Extent of Electroporation Effects and BBB Disruption

For a given HF-PEF waveform, namely the 2-5-2 μ s, line plots of the electric field magnitudes were evaluated to (1) bisect the monopolar probes (Figure 3b); (2) bisect the bipolar probe (Figure 3c); and (3) to bisect the single-needle grounding pad (Figure 3d). From this line plot, the EFTs were used to map the cell electroporation effects and BBBd to demonstrate the propensity of each electrode configuration to maximize inducing large reversible and irreversible electroporation effects (monopolar probe) and to maximize large BBBd (single-needle grounding pad). These data show that while keeping the HF-PEF waveform constant, the electrode configuration employed can affect the extent of BBBd, ablation, reversible electroporation, and nerve excitation.

In a similar analysis, these effects are compared not only across electrode configurations but across the 2-5-2 μ s and the 5-5-5 μ s waveforms (see supplementary Figure S5). In this case, it is apparent that the monopolar probe electrode configuration implemented with a 5-5-5 μ s waveform is amenable for ablation therapy. This implementation demonstrated numerical ablation, reversible electroporation, BBBd, and nerve excitation as the total widths (2X the values of Figure S5) 2.08 cm, 2.29 cm, 4.27 cm, and 7.29 cm, respectively. Contrarily, the single-needle + distant grounding pad electrode configuration implemented with a 2-5-2 μ s waveform constitutes a minimally invasive, large-volume BBBd therapy. With a comparable nerve excitation dimension, this implementation demonstrated numerical ablation, reversible electroporation, BBBd, and nerve excitation as the total widths (2X the values of Figure S5) 0.57 cm, 1.06 cm, 4.94 cm, and 7.71 cm, respectively. Specifically, this BBBd protocol capitalizes on the short pulse width to reduce ablation and further capitalizes on the diffuse electric field distribution for large volume BBBd. More notably, the single-needle approach is capable of producing large radially symmetric distributions which are often desired when treating irregular shapes. These results are visual demonstrations; a quantification of these effects is carried out to also include the effects of the HF-PEF waveform.

#### 2.5.2. HF-PEF Waveforms Selectively Modulate the Extent of Electroporation Effects, BBB Disruption, and Nerve Excitation

**Figure 5:**
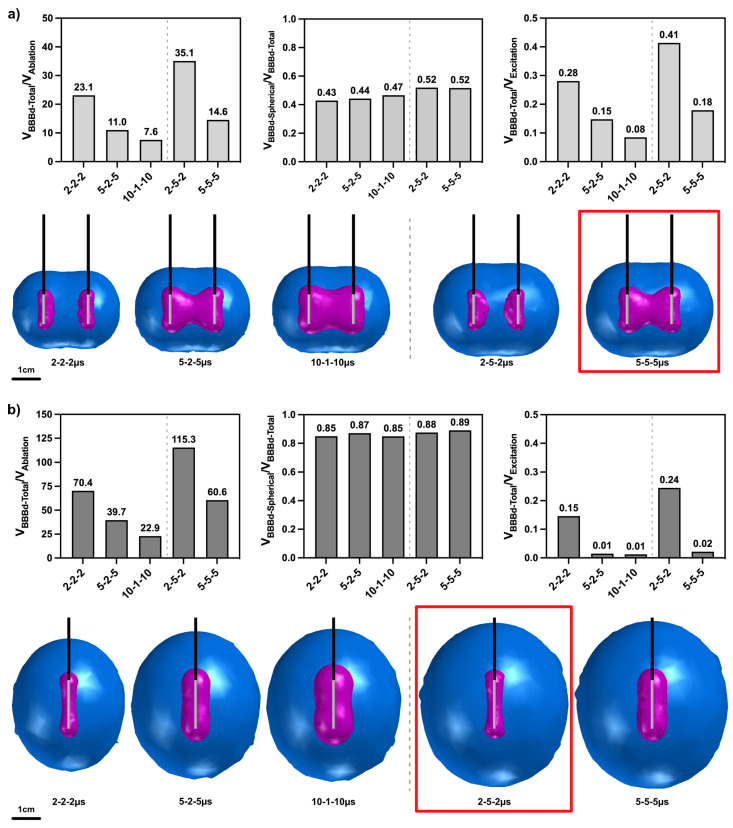
Pulse width and interphase delay modulate the ratio of BBB disruption volume to cell ablation. For the (**a**) monopolar probe and the (**b**) single-needle grounding pad configuration, ratios of BBB disruption volume to ablation, largest encapsulated spherical BBB disruption to total BBB disruption volumes, and BBB disruption volume to nerve excitation volume were quantified. Three-dimensional contours across waveforms and configurations were generated to scale to directly compare ablation (magenta) and BBB disruption (blue) volumes. In summary, the monopolar probes are amenable to large ablations (outlined in red box) while the diffuse electric field distribution of the single-needle grounding pad maximizes BBB disruption effects (outlined in red box). Lower pulse widths and higher delays maximize BBB disruption effects, whereas lower pulse widths reduce nerve excitation and ablation.

For the three electrode configurations, the five HF-PEF waveforms were evaluated and volumes of BBBd, ablation, reversible electroporation, and nerve excitation were quantified based on their respective EFTs. Figure 5 demonstrates the size and shape of ablations (magenta) and BBBd (blue) predicted for a 2 kV treatment for the monopolar and single-needle grounding pad configurations across the five HF-PEF waveforms. Comparison was made for the total BBBd volume (V_BBBd-Total_), total ablation volume (V_Ablation_), volume of the largest sphere encapsulated within V_BBBd-Total_ (V_BBBd-Spherical_), and the volume susceptible to nerve excitation (V_Excitation_). Ratios for these values are shown in Figure 5. Notably, for a given interphase delay (either 2 μ s or 5 μ s), a decrease in the pulse width increases the V_BBBd-Total_/V_Ablation_ ratio as well as the V_BBBd-Total_/V_Excitation_ ratio. An increase in interphase delay also increases these two ratios. This analysis is also conducted for the bipolar probe configuration and is seen in the supplementary materials (Figure S6). Lastly, nerve excitation, BBB disruption, and cell ablation effects are plotted for direct visual comparison between HF-PEF waveform and electrode configuration and are seen in the supplementary materials (Figure S7).

To determine the pulsing parameters and the electrode configurations for maximizing favorable BBBd, minimizing electroporation effects, and minimizing nerve excitation, we introduce the dimensionless term ζ to denote the “relative efficiency” of the treatment protocol:

(1) $$\zeta =A_{1}\cdot \frac{V_{\mathrm{BBBd-Total}}}{V_{\mathrm{Ablation}}}+A_{2}\cdot \frac{V_{\mathrm{BBBd-Spherical}}}{V_{\mathrm{BBBd-Total}}}+A_{3}\cdot \frac{V_{\mathrm{BBBd-Total}}}{V_{\mathrm{Excitation}}}$$

Equation (1) is divided into three components that collectively identify a pulsing protocol and electrode configuration for maximizing the BBBd volume while minimizing the ablation volume and volume susceptible to nerve excitation. The first component, $V_{\mathrm{BBBd-Total}}/V_{\mathrm{Ablation}}$ , quantifies the ability to minimize the total ablation induced by a particular protocol while maximizing the total BBBd induced. Often, a spherical region of BBBd is desired, therefore a second term assessing a type of sphericity is created. We designated the largest sphere that fits within the total BBB volume to be this desired volume. The ratio $V_{\mathrm{BBBd-Spherical}}/V_{\mathrm{BBBd-Total}}$ quantifies the clinical relevance of a spherical BBBd region. Finally, diffuse field gradients benefit from large BBBd regions but are also susceptible to propagating low fields which induce nerve excitation (Figure 3). In an effort to minimize the significant contributions of nerve excitation, we considered $V_{\mathrm{BBBd-Total}}/V_{\mathrm{Excitation}}$ . To make direct comparisons across both the waveforms and the electrode configurations, each component of the relative efficiency Equation (raw values are seen in Figure 5) was normalized to the absolute maximum value of the respective ratio across all configurations and waveforms. Thus, each term was scaled from a value 0 to 1, and weighting factors were used to balance this equation.

**Figure 6:**
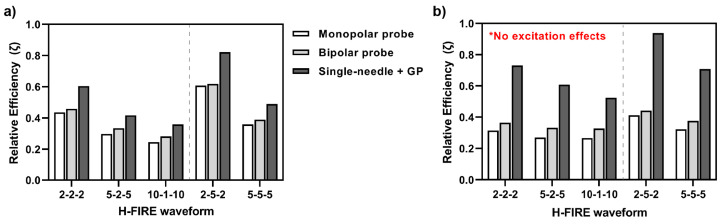
A non-dimensional relative efficiency term (Equation (1)) was defined to transcend and normalize BBBd, ablation, and nerve excitation effects across all waveforms and electrode configurations: (**a**) for a scenario where a neuroparalytic is not administered, all components of Equation (1) are equally weighted at A_1_ = A_2_ = A_3_ = 0.33, resulting in a significant advantage of the 2-5-2 μ s waveform for maximizing all desirable effects; (**b**) for a scenario where a neuroparalytic is administered, all contributions of nerve stimulation in our efficiency terms are nullified (A_1_ = A_2_ = 0.5, A_3_ = 0), further highlighting the advantage of the single-needle grounding pad configuration to maximize BBB disruption and minimize ablation effects.

Two scenarios of this equation were solved. In the first scenario, the weighting coefficients were equally weighted (A_1_ = A_2_ = A_3_ = 0.33). Inherently, this scenario implies a situation in which a neuroparalytic is not administered and excitation effects are considered. These results are seen in Figure 6a, where, despite the single-needle grounding pad showing the largest V_BBBd-Total_/V_Ablation_ ratio, the effects of excitation make it comparable to the relative efficiency of the monopolar probe and bipolar probe. Notably, even when excitation effects are considered, the 2-5-2 μ s waveform demonstrates the highest overall relative efficiency value.

In a second scenario (Figure 6b), the weighting coefficients are not equally weighted and take on values A_1_ = A_2_ = 0.5, A_3_ = 0. Specifically, this term disregards nerve excitation effects on the relative efficiency, implying this is a scenario in which a neuroparalytic is administered and emphasis is placed on BBBd effects. Here, the single-needle grounding pad takes on relative efficiency values higher than those of the monopolar probe and the bipolar probe. The results of Figure 6 highlight the 2-5-2 μ s HF-PEF waveform across all configurations in scenarios both without (a) and with (b) the administration of a neuroparalytic. For intracranial clinical surgeries where a paralytic is administered, the single-needle grounding pad configuration implemented with 2-5-2 μ s waveforms will result in the highest BBBd, most spherical BBBd geometry, and minimal ablation.

## 3. Discussion

Primary brain tumors often have a poor prognosis due to the combination of their infiltrative nature and the inability of therapeutic drugs to penetrate the tumor mass due to the presence of the BBB. In particular, the brain tumor glioblastoma is the leading cause of cancer-related death in children under 19, with a mean survival of fewer than 15 months. As suggested by the results in this study, the utility of BBB disruption for brain tumors, specifically for that of HF-PEF mediated BBBd, is such that large volumes (>3 cm spherical diameter) of BBBd can be induced in a relatively short amount of time (~200 s). Other therapies take advantage of BBBd for the clearance of protein aggregates which were found to correlate with the incidence of Alzheimer’s disease, as well as neuromodulation for diseases such as Parkinson’s and epilepsy. Traditionally, HF-PEF BBBd is used to induce a focal lesion with peri-ablative BBBd. Having a better understanding of the contribution of parameters like pulse width and interphase delay may help maximize the efficacy of protocols where a lesion is not desired but a large BBBd is necessitated for targeting large brain tumors and other neurological conditions. Applying an ablation maximization protocol may be desirable if directly pulsing into a large tumor; however, a BBBd maximized protocol for the surrounding infiltrative tissue may allow for more diffuse treatment when combined with other therapeutics.

In this study, we investigated the contributions of both the HF-PEF waveform and the electrode configuration to modulate the extent of BBBd, reversible electroporation, cell ablation, and nerve excitation using a combination of in vivo, in vitro, and in silico methods. The single-needle grounding pad configuration provides a narrow window (<~0.5 cm) of high electric fields adjacent to the electrode to induce cell death. However, the diffuse field distribution maximizes regions of lower strength fields capable of inducing BBBd > 3 cm in a spherical diameter. Compared to the dual monopolar configuration, which induces larger proportions of ablation-inducing fields, this single-needle configuration provides more comprehensive coverage of the BBBd and produces smaller ablations. Additionally, this configuration is less intrusive by only requiring the insertion of a singular needle as opposed to two or more, thereby reducing the cranial defect size required for needle placement. This may further reduce OR time and prevent clinician errors by eliminating the possibilities of electrode skewness and angulation during the insertion process. Field distributions generated by monopolar and bipolar electrode configurations are often asymmetric and even discontinuous when the applied voltage is not significant. On the contrary, both high and low electric fields produced from the single-needle grounding pad approach are more spherical and thus may be more desirable.

Prioritizing increased BBBd volumes presents an opportunity to enhance the pharmacological treatment of large brain tumors as lower voltages are required to reach clinically relevant BBBd volumes (>3 cm spherical diameter). Ablative lesions require high voltages for the adequate coverage of the tumor; if electrochemical effects or other factors limit the voltage that can be applied, the periphery of the tumor may go untreated. The transition to H-FIRE from previous-generation IRE, which utilized much greater pulse widths (>50 μ s), should help to alleviate these effects. With large BBBd, the opportunity to use the therapy concomitantly with a drug or chemotherapeutic to eradicate infiltrative disease is promising. As HF-PEFs mediated BBBd is a transient process [36], Lorenzo et al. demonstrated that the BBBd lifetime induced by HF-PEFs [18] is 72 h and recovers to ~50% after 24 h. The effects of prolonged BBB disruption may result in hemorrhaging, inflammation, and edema in the brain parenchyma. An investigation of the safety and efficacy of long-lived BBBd is certainly warranted, though outside the scope of this study.

From our results, it is evident that BBBd occurs at electric fields far below reported thresholds of electroporation. We report minor differences in BBBd thresholds across waveforms with a maximum difference of 82.7 V/cm and differences in ablation thresholds of up to 519.7 V/cm. The mechanism of BBBd is suggested to be linked to the disruption of the tight junction proteins [19], making this phenomenon physiologically distinct from the mechanisms of electroporation. Taking advantage of this threshold gap, we can implement a BBBd maximization protocol by utilizing the waveform with the most considerable differences in these thresholds.

Simulations investigating the effects of waveform concluded that the 2-5-2 μ s waveform was consistently superior for maximizing BBBd volumes while minimizing ablation volumes and nerve excitation. More generally, larger pulse widths correlated with larger ablation volumes, whereas increasing the interphase delay improved BBBd effects, creating an opportunity for HF-PEFs to capitalize on lower magnitude fields for inducing BBBd. By increasing the interphase delay, the characteristic frequency of the given waveform decreased and the nerve excitation threshold decreased, risking more pronounced nerve stimulation and muscle contraction. Whether differences in nerve excitation from increasing the interphase delay are sufficient to warrant concern is unknown, though future experiments can focus on these effects. While the presented data support the use of the single-needle grounding pad configuration and the 2-5-2 μ s waveform as a BBBd protocol, it is important to note that the single-needle grounding pad configuration heightens the risk for nerve excitation. A notable advantage of H-FIRE compared to its first-generation counterpart, IRE, is the proclivity to mitigate the extent of muscle contractions due to short pulse widths, short interphase delays, and bipolar pulses employed to prevent the production of action potentials [29]. Nonetheless, conditions exist where the need for a neuroparalytic is still plausible and even desired for even larger BBBd volumes using higher voltages.

A limitation of our study includes that our reversible electroporation and ablation thresholds were not validated in vivo. However, it is well established in tissue engineering and cancer biology literature that cells cultured within a 3D hydrogel scaffold grow into relevant phenotypes and respond to stimuli in a manner comparable to that observed within in vivo models [38,39,40]. In future work, it may be useful to validate our thresholds in an in vivo model. In this study, only a snapshot of the BBBd was quantified. In all cases, MRI and gross pathology results were conducted and represent the state of the tissue 1 h following treatment with HF-PEFs, rather than quantifying the dynamics of BBBd as previously achieved [36]. Another limitation that falls within our numerical simulations includes the absence of the skull and skin domains in an effort to simplify the model. Although we expected a loss of potential across these domains, we did not expect it to significantly impact our distributions. Should the effects be significant, one may overcome these effects by moderately increasing the applied voltage. This then brings into conversation the design and functional requirements of pulse generators to deliver this therapy [41]. As BBBd is induced at much lower electric fields compared to IRE and reversible electroporation, existing generators may easily be adapted for PEF-BBBd therapy. In clinical applications, the location and size of the grounding pad will also affect the muscle excitation experienced. In this case, a paralytic can be administered, or the grounding pad placed in a desired location. Additionally, rodents in our study underwent craniectomies for the insertion of needle electrodes into the brain, making this a minimally invasive procedure. Alternatively, there is the potential for a non-invasive BBBd approach using pulsed electromagnetic fields (PEMFs) [42]. While promising, a technical challenge facing PEMF-mediated BBBd may include focusing the EMFs to create focal permeabilization to limit off target effects. One such approach to address this challenge may include the use of conductive nanoparticles to locally enhance the effects of EMFs [43,44].

In summary, we reported the treatment conditions resulting in PEF protocols amenable to either large BBBd (highest relative efficiency, Figure 6) or maximizing ablation effects (monopolar probe, longer pulse widths, Figure 5). A significant conclusion of this study is that, in all cases, including in the presence and absence of a neuroparalytic, the single-needle grounding configuration implemented with a 2-5-2 μ s waveform is the most relatively effective at maximizing the BBBd-to-ablation ratio and inducing spherical BBBd volumes while taking into consideration the contributions of nerve excitation.

## 4. Materials and Methods

### 4.1. Surgical Procedures and Assurances

This study was performed in parallel to that of Lorenzo et al. [18] and was conducted in accordance with the principles of the Guide for the Care and Use of Laboratory Animals and was approved by the Institutional Animal Care and Use Committee (IACUC#16-156). Briefly, 18 male Fischer rats weighing between 170 and 215 g were premedicated with a subcutaneous (1 mg/kg) injection of buprenorphine (Buprenorphine SR-LAB; Zoopharm, Windsor, CO, USA), anesthetized using isoflurane induction (3–4%:95% isoflurane:oxygen mixture), and then maintained with isoflurane (2–3.5%:95% isoflurane:oxygen mixture) delivered via nose cone. The dorsum of the head from the intercanthal area to the cranial cervical region was clipped and prepared for aseptic surgery. Anesthetized rats were instrumented in a small animal stereotactic headframe (Model 1350 M; David Kopf Instruments, Tujunga, CA, USA). A unilateral rostrotentorial surgical approach to the skull was performed and a 5 mm × 2.5 mm rectangular, parietal craniectomy defect was created in the skull of each rodent using a high-speed electric drill (Dremel 3000 Series; Mount Prospect, IL, USA) with a 2.4 mm diameter and round burr. Thereafter, two blunt-tipped stainless-steel electrodes were advanced into the brain using the micromanipulator arm of the stereotactic frame according to stereotactic coordinates referenced to the location of the rostral electrode (bregma 4 mm caudal, 3.5 mm lateral, at a depth of −4 mm relative to the surface of the dura).

Pulse delivery commenced according to the pulsing parameters defined in Table 1. Following treatment, the electrodes were retracted, the craniectomy defect was covered with bone wax (Ethicon, Somerville, NJ, USA), and the skin incision was closed with 4-0 monocryl interrupted skin sutures (Ethicon). Rats were recovered from anesthesia and monitored until the 1 h predetermined survival endpoint.

### 4.2. High-Frequency Pulsed Electric Fields and Parameter Selection for In Vivo Studies

A custom bipolar pulse generator (VoltMed Inc, Blacksburg, VA, USA) was used to deliver HF-PEFs. This generator is capable of producing voltage waveforms in a bipolar manner with a maximum voltage/current output of 5 kV/100A. Voltage and current waveforms were recorded using a WaveSurfer 3024 z oscilloscope (Teledyne LeCroy, Chestnut Ridge, NY, USA) with a 1000X high voltage probe (Enhancer 3000, BTX, Holliston, MA, USA) and 10X current probe (2877, Pearson Electronics, Palo Alto, CA, USA) as seen in Figure S1. The electrodes ( ϕ = 0.45 mm) were fixed at a center-to-center spacing of 4 mm, and a thin polyimide sheath was used such that the electric fields were focused on the exposed tips with a 1 mm electrode exposure.

The nomenclature to define the burst of biphasic PEFs is: positive phase–interphase delay–negative phase μ s (Figure S1). The pulse width and the interphase delay were varied, though for each treatment, the burst on-time was maintained at 100 μ s, 200 bursts were delivered, and the applied V/d ratio was constant at 600 V/cm (240 V applied).

### 4.3. MR Imaging and Gross Pathology for In Vivo BBB Disruption Volumetrics

BBB permeability was measured via contrast-enhanced T1W MRI and in gross pathology via Evans blue dye staining; a more detailed analysis was found in Lorenzo et al. [18]). An intraperitoneal injection of 0.1 mmol/kg of gadopentetate dimeglumine (Gd; Magnevist; Bayer, Whippany, NJ, USA) and 75 mg/kg of 2.5% Evans blue dye (EBD; Sigma; St. Louis, MO, USA) was administered 5 min prior to HF-PEFs treatment. The Gd-EBD solution was administered 1 h prior to sacrifice to allow for the diffusion and uptake from systemic circulation into regions where the BBB is disrupted. The anesthetized rats were euthanized by IP pentobarbital (0.5 mL) overdose (Fatal Plus, Vortech Pharm, Dearborn, MI, USA). MRI images of the brain were acquired immediately after euthanasia with a Philips 1.5 T scanner (Intera, Philips Healthcare, Andover, MA, USA) equipped with an 8-channel head coil. The T1-weighted spin-echo was acquired using the given parameters: repetition time (TR) = 450 ms, echo time (TE) = 15 ms, field of view (FOV) = 40 mm, and slice thickness = 2.0 mm.

Following MRI imaging, the brain of each rodent was removed and immersion-fixed in 10% neutral buffered formalin solution. Following fixation for 48 h, the brain of each rodent was placed in an adult rodent matrix slicer (Ted Pella Inc., Redding, CA, USA), serially sectioned in the coronal plane at 2 mm intervals, and individually paraffin embedded in a tissue cassette. Coronal brain sections containing EBD were serially sub-sectioned in the coronal plane at 10 µm thickness and 200 µm intervals using a microtome and mounted on positively-charged microscope slides. Digital photomicrographs (Nikon Eclipse Ni-E, Nikon, Japan) of the intraparenchymal EBD were obtained from all intervening coronal sections using a charge-coupled device camera with a fixed aperture (Nikon DS-Fi1c, Nikon, Japan). The volume of EBD resulting from the coronal image stack from each rat was calculated using a Cavalieri estimator on a commercial image analysis system (Stereo Investigator; MBF Bioscience, Williston, VT, USA). Finally, EBD fluorescence in brain tissue and blood samples was processed using a previously described dye extraction method (see Supplementary Material for details) [45].

### 4.4. Cell Culture and Determination of H-FIRE Ablation Electric Field Thresholds

Healthy rodent astrocytes (DI TNC1; ATCC, Manassas, VA, USA) were incubated at 5% CO_2_ and 37 °C in Dulbecco’s modified Eagle medium (DMEM; Sigma Aldrich, St. Louis, MO, USA) supplemented with 1% penicillin/streptomycin (Life Technologies) and 10% fetal bovine serum (Atlanta Biologicals, Atlanta, GA, USA). Cell lines were passaged at 80–90% confluency.

A 3D collagen hydrogel tissue mimic was constructed for in vitro investigations of cell ablation and cell reversible electroporation in DI TNC1 cells [38]. Commercial rat collagen (BD Biosciences, Franklin Lakes, NJ, USA) and collagen extracted in-lab (see Supplemental Material) were utilized. With either type of collagen, a neutralizing buffer solution consisting of 10× DMEM (10% total volume; Sigma Aldrich, St. Louis, MO, USA), 1N NaOH (2% total collagen volume; Sigma Aldrich), and 1× DMEM was used to achieve a final concentration of 5 mg/mL. Cells were trypsinized, counted, and resuspended within the 1× DMEM introduced into the buffer, at a concentration of 1.5 × 10^6^ cells/mL. The mixture of collagen and cells was dispersed into culture-treated 12-well plates at a 1 mm thickness and stored in a cell culture incubator to allow for polymerization. After 25 min, 500 μ L of DMEM was supplemented to each collagen hydrogel, which were returned to incubation for 24 h until treatment.

For HF-PEF treatment, media was aspirated from each well and a single-needle ( ϕ _OD_ = 1.65 mm) and grounding ring ( ϕ _ID_ = 1.8 cm) configuration (Figure 2a,b) was used to produce circular regions of ablation and reversible electroporation. Pulses were delivered using a custom pulse generator (VoltMed Inc, Blacksburg, VA, USA) and the waveforms were monitored using an oscilloscope equipped with a 1000X high voltage probe and a 10X current probe. Treatment consisted of 100 bursts delivered at a voltage amplitude of 600 V, with each burst energized for 100 μ s. The five bipolar waveforms were investigated and are listed in Table 2. Cell death thresholds were extracted 24 h after treatment to allow for the complete recovery of reversibly electroporated cells (Figure 2), while reversible thresholds were analyzed 45 min after treatment as determined from a time-lapse study (Figure S2).

Ablations were evaluated using fluorescence microscopy through live/dead staining with a solution consisting of 2.5 μ M of calcein AM (Invitrogen, Carlsbad, CA, USA) and 15 μ M of propidium iodide (Invitrogen) in phosphate-buffered saline (PBS). The collagen hydrogels were either stained 30 min prior to pulsing for the reversible treated groups or stained 24 h after treatment as previously mentioned. Hydrogels stained 24 h after treatment underwent two washes of PBS before imaging while the hydrogels stained prior to treatment were not washed with PBS, but aspirated until a thin layer was remaining to coat the hydrogel. The viability was assessed using both a Leica fluorescence microscope (DMI 6000 Leica Microsystems, Buffalo Grove, IL, USA) and Zeiss LSM 800 confocal microscope (Carl Zeiss Microscopy LLC, Thornwood, NY, USA) under 5× magnification and were analyzed using ImageJ.

### 4.5. Numerical Determination of BBBd, Ablation, Electroporation, and Nerve Excitation Thresholds

To determine the BBB disruption field thresholds, a numerical model was constructed using COMSOL Multiphysics v5.6 (COMSOL Inc., Stockholm, Sweden) and a rat brain tissue domain was imported following segmentation from a T1W MRI scan using 3D Slicer 4.10 (Slicer, https://www.slicer.org/, accessed on 13 October 2019). The final domain, including the brain and two monopolar electrodes with insulation, consisted of 298,218 tetrahedral elements resulting from an “extra fine” mesh setting. After mesh generation, the electric potential distribution was modeled using Equation (2):

(2) ∇·(σ∇ϕ)=0

where ϕ is the electric potential and σ is the tissue conductivity. Electrical and thermal properties for the brain tissue domain, insulation domain, and the stainless steel electrode domain are included in Table 3 and are taken from either the COMSOL material library or from the IT’IS Dielectric Properties database [46]. Electroporation effects in vivo are known to increase the electrical conductivity as a function of the local electric field [47]. This electroporation effect can be approximated using a sigmoid relationship (Equation (3)) [48] and is coupled to the increase in conductivity due to Joule heating (Equation (4)):

(3) $$\sigma \left(E\right)=\sigma_{0}\cdot \left(1+A\cdot flc2hs\left(E-E_{delta},E_{range}\right)\right)$$

(4) σ(E,T)=σ(E)·(1+α·ΔT)

The conductivity sigmoid takes on an initial value $\sigma_{0}$ , representing tissue conductivity in an un-electroporated state, and plateaus to a value of $\sigma_{f}$ , representing tissue conductivity in an electroporated state. In this equation, the value $\sigma_{f}$ is related to parameter A by $\sigma_{f}$ = $\sigma_{0}\cdot \left(1+A\right)$ . The sigmoid transitions at $E_{delta}$ over a range ± $E_{range}$ . The parameter $\sigma_{0}$ varies for each HF-PEF waveform and was taken as the conductivity of gray matter tissue (Gabriel et al. [49]) at a frequency equal to the characteristic frequency of each waveform (Table 4). The parameter $\sigma_{f}$ was identical for all waveforms and was taken as the conductivity of gray matter tissue (Gabriel et al. [49]) at a frequency of 10 MHz, as high-frequency impedance measurements reduce the cell membrane reactance and take on values similar to the impedance of electroporated tissues [50]. The parameter E_delta_ has been shown to relate to the lethal EFT [51], therefore this parameter varies with the HF-PEF waveform and was assumed to be equal to the lethal EFT for each waveform. Lastly, the parameter E_range_ has not been extensively investigated, therefore, a value of 350 V/cm was selected based on studies from Zhao et al. [48]. These conductivity sigmoids were plotted as seen in the supplementary materials (Figure S8). Boundary conditions were set to mimic in vivo treatment, where one electrode was set to a potential boundary condition of ϕ = 240 V and the other electrode was set to a ground, ϕ = 0 V. All remaining external boundaries were assumed to be electrically insulating.

Joule heating and thermal conduction were accounted for by incorporating a modified bioheat equation:

(5) $$\rho c_{p}\frac{\partial T}{\partial t}=\nabla \cdot \left(k\nabla T\right)-\omega_{b}\rho_{b}c_{b}\left(T-T_{b}\right)+\frac{\sigma \cdot {\left|E\right|}^{2}\cdot p}{\tau }$$

where ρ is the tissue density; c_p_ is the specific heat; k is the thermal conductivity; $\omega_{b}$ is a distributed blood perfusion coefficient; $\rho_{b}$ is the blood density; *c_b_* is the specific heat of blood; and *T_b_* is the temperature of blood. In this study, *p* is the burst on-time (100 $\times \;10^{-6}$ s) and τ is the period of burst delivery (1 s). These terms represent a duty cycle approach, which allows for thermal contributions from high-frequency PEFs to be modeled as a continuous heat source rather than a periodic heat source.

BBBd EFTs were numerically determined as the electric field contour which encapsulates the same volume of tissue as the BBBd volume from gross pathological tissue sections. This analysis makes the assumption that the measured BBBd was topologically consistent with computed field contours.

Extraction of ablation and reversible electroporation thresholds was identical to that of the in vivo rodent brain model, though here a hydrogel domain (1 mm thick, 1.8 cm diameter cylinder), grounding ring domain (ID = 1.8 cm, OD = 2.0 cm, height = 2.5 cm), and needle electrode (ID = 1.35 mm, OD = 1.65 mm, height = 2.5 cm) domain were used. The electrical conductivity of the stainless steel was that shown in Table 3, though the conductivity of the hydrogel was constant and set to σ _hydrogel_ = 1.8 S/m. A temperature coefficient of conductivity, α _hydrogel_ = 2 %/°C, was used. Boundary conditions were set to mimic the in vitro treatment, where the single-needle was set to a potential boundary condition of ϕ = 600 V and the grounding ring was set to a ground, ϕ = 0 V. The final domain consisted of 237,613 tetrahedral elements.

Effects of nerve excitation were modeled through a modified SENN nerve fiber model [29,37]. As electroporation treatments are conventionally performed with needle electrodes, a nerve terminus was modeled in the vicinity of the electrodes and in parallel with a given electric field contour. The model simulates a 6-node myelinated nerve fiber terminus exposed to a uniform electric field with the given temporal characteristics. For a given waveform, the applied electric field was increased in increments of 0.25% until an action potential was generated within the nerve fiber. Thresholds determined from this model can be used in clinically relevant computations to approximate the region around the electrodes within which such a fiber would be excited. A custom MATLAB (MathWorks Inc., Natick, MA) script was written to model waveforms in Table 2, and a summary of the nerve excitation threshold can be found in Section 2.4.

### 4.6. Numerical Methods for Elucidating the Effects of HF-PEF Waveforms on BBB Disruption, Cell Ablation, Electroporation, and Nerve Excitation

Following the extraction of EFTs, a 3D finite element model was developed in COMSOL Multiphysics to simulate the effects of HF-PEF waveform on BBBd, ablation, reversible electroporation, and nerve excitation in representative clinical settings. This method of numerical analysis has been shown to precisely predict field distributions correlating with both reversible and irreversible electroporation in vivo [52]. Like the model in Section 4.5, Equation (2) was used to solve the electric potential distribution. The effects of HF-PEF waveforms were modeled in two ways: (1) the electrical conductivity sigmoid was specific for each waveform, and (2) the electric field threshold for BBBd, ablation, reversible electroporation, and nerve excitation were specific for each waveform. The electrical conductivity of gray matter was modeled using the sigmoid in Equation (3) (and parameters in Table 4 for each waveform) and Joule heating effects coupled using Equation (4). Additional electrical and thermal properties are reported in Table 3.

Three distinct electrode configurations were modeled; this included the dual monopolar probe configuration, the single insertion bipolar probe configuration, and the single-needle + distant grounding pad configuration (Figure 4). Both the monopolar probe and bipolar probe configurations have been clinically used for the IRE ablation of various tumor types [53,54], whereas the single-needle grounding pad configuration was preclinically tested [55].

The dual monopolar probes were modeled as 1 mm diameter cylinders with a height of 1 cm and center-to-center spacing of 1.5 cm. A single-insertion bipolar probe was modeled as three co-linear cylinders, with the two exposed metal electrodes measuring 1.6 mm in diameter and 7 mm in height, separated by an 8 mm insulator. The single-needle grounding pad was set up as a 1 mm diameter cylinder with a height of 1.7 cm and a distance of 30 cm from the grounding pad (20 cm diameter circular pad with 1 mm thickness). All electrodes were then assigned a long insulating handle. The electrode exposures in each configuration were modeled as stainless steel cylinders and the electrode handles and bipolar separation were modeled as an insulator (Table 3). Electric potential ( ϕ = 2 kV), ground ( ϕ = 0 V), and insulation boundary conditions were applied to their corresponding electrode and external boundaries, respectively. Finally, the gray matter brain tissue domains for the monopolar probe and bipolar probe configurations were modeled as 20 cm in diameter and 20 cm in height to avoid boundary effects on the electric field distribution. The field distribution for the single-needle grounding pad was more diffuse; therefore, the cylindrical tissue domain was increased to 80 cm in diameter and 60 cm in height to prevent boundary effects. It is important to note that these tissue domains were not intended to represent the geometry of the brain, but rather to model electric fields in a domain with no boundary effects. The total number of elements in the final mesh was 314,170 elements for the monopolar probe simulation, 265,276 for the bipolar probe simulation, and 1,240,185 for the single-needle grounding pad simulation, respectively, corresponding to a modified “extra fine” mesh setting in COMSOL.

A batch sweep process was used to run simulations in parallel across the available cores in the CPU. Once solved, volume integrations for electric field magnitudes greater than or equal to the BBBd, ablation, reversible electroporation, and nerve excitation thresholds were implemented to solve for volumetrics. As shown in Figure 5, the volumes of total BBBd (V_BBBd-Total_), ablation (V_Ablation_), and nerve excitation (V_Excitation_) were quantified. In addition, an analysis to solve for the “largest spherical BBBd volume” contained within the total BBBd was quantified. This metric (V_BBBd-Spherical_) is meant to inform the utility of the BBBd from the three different electrode configurations. The ratios V_BBBd-Total_/V_Ablation_, V_BBBd-Spherical_/V_BBBd-Total_, V_BBBd-Total_/V_Excitation_ were calculated and compared across the HF-PEF waveforms and across electrode configurations, as seen in Figure 5. Finally, for direct comparison of BBBd and ablation capacity of all waveforms and electrode configurations, a non-dimensional parameter, termed “Relative Efficiency” (Equation (1)), was calculated. This equation multiplies weighting coefficients to each of the three ratios. In Figure 6a, the weighting coefficients are equally weighted (A_1_ = A_2_ = A_3_ = 0.33). Specifically, this analysis is weighing for maximizing the V_BBBd-Total_/V_Ablation_ ratio, maximizing for the BBBd with the least off target effects (most spherical geometry), and maximizing V_BBBd-Total_/V_Excitation_ to reduce nerve excitation effects. Inherently, this scenario implies a situation in which a neuroparalytic is not administered. In Figure 6b, the weighting coefficients are not equally weighted (A_1_ = A_2_ = 0.5, A_3_ = 0). Specifically, this term disregards nerve excitation effects on the relative efficiency, implying a scenario in which a neuroparalytic is administered and emphasis is only placed on BBBd effects.

### 4.7. Statistical Analysis

Statistical analysis was conducted in GraphPad Prism v9.3. A Kruskal–Wallace test with an uncorrected Dunn’s multiple comparisons was used for the comparison of the HF-PEF waveform on in vivo BBBd volumetrics. Pairwise comparison for MRI vs. EBD volume BBBd measurements was accomplished using a Wilcoxon matched-pairs signed-rank test. To compare the results of all waveforms without pairing for pulse width or interphase delay, including comparison to the 10-1-10 μ s waveform, a one-way ANOVA with Bonferroni’s multiple comparison was used. In addition, a two-way ANOVA with Bonferroni’s multiple comparison was used to compare the effects of pulse width and interphase delay (2 μ s vs. 5 μ s). In this analysis, the 10-1-10 μ s waveform was omitted from comparison. In all cases, a *p*-value < 0.05 was considered the threshold for statistical significance.

## 5. Conclusions

High-frequency pulsed electric fields have shown promise in their ability to induce the disruption of the blood-brain barrier. This investigation provides valuable data regarding the contributions of waveform selection and electrode configuration. Both in vivo and in vitro experiments were able to provide waveform-specific electric field thresholds for inducing BBB disruption, reversible electroporation, and ablation, a collection of data not previously presented as a whole. It was found that increasing the pulse width correlated with larger ablation distributions, while larger interphase delays correlated with larger volumes of BBB disruption. Incorporating these data into numerical models, it was determined that the single-needle grounding pad configuration most effectively generates a diffuse BBB disruption volume that is clinically relevant, in addition to minimizing ablation. While the diffuse field distribution of the single-needle grounding pad configuration leads to enhanced volumes of nerve excitation, models predict that the 2-5-2 μ s waveform can largely reduce muscle contractions while still maintaining the efficacy of achieving large BBB disruption volumes. While further in vivo data are needed to validate these studies, the framework presented in this paper shows promise for improving outcomes in BBB disruption-based therapies.

## 6. Patents

Melvin F. Lorenzo, Sabrina N. Campelo, John H. Rossmeisl, Jr., Christopher B. Arena, and Rafael V. Davalos have filed an IP disclosure on the work discussed in this article. Melvin F. Lorenzo, Kenneth N. Aycock, Christopher B. Arena, John H. Rossmeisl, Jr., and Rafael V. Davalos have issued patents and/or patents pending in the area of irreversible electroporation and may receive royalties.

## Figures and Tables

**Table 1:** Quantification of in vivo BBB disruption by CE MRI, gross pathology, and EBD fluorescence (mean ± SD).

Waveform	Pathological BBBd (mm^3^)	MRI BBBd (mm^3^)	Cerebral (EBD) ( μ g/g)	Serum (EBD) ( μ g/g)
Sham	0.0 ± 0.0	0.0 ± 0.0	0.2 ± 0.0	1494.0 ± 0.0
2-2-2 μ s	36.6 ± 9.4	36.7 ± 13.0	14.1 ± 0.2	1532.8 ± 137.5
5-2-5 μ s	53.9 ± 8.1	59.2 ± 10.8	15.2 ± 0.1	1363.3 ± 152.4
10-1-10 μ s	61.0 ± 2.8	60.0 ± 4.2	N/A	N/A
2-5-2 μ s	74.1 ± 7.8 ^**^	74.7 ± 9.8 ^*^	16.9 ± 0.1	1326.0 ± 24.7
5-5-5 μ s	81.2 ± 8.0 ^**^	84.1 ± 8.7 ^**^	18.5 ± 0.3 ^*^	1318.3 ± 66.8

Comparison of HF-PEF waveform and the sham of each column, respectively. * denotes a *p*-value ≤ 0.05 and ** denotes a *p*-value ≤ 0.01.

**Table 2:** Summary of in vivo BBB disruption and in vitro ablation and reversible electroporation electric field thresholds.

Waveform	Pathological BBBd (mm^3^)	BBB Disruption Threshold (V/cm)
2-2-2 μ s (n = 4)	36.6 ± 9.4	216.5 ± 32.7
5-2-5 μ s (n = 4)	53.9 ± 8.1	183.0 ± 18.0
10-1-10 μ s (n = 2)	61.0 ± 2.8	178.0 ± 6.0
2-5-2 μ s (n = 4)	74.1 ± 7.8	133.8 ± 11.4
5-5-5 μ s (n = 4)	81.2 ± 8.0	136.3 ± 10.0
**Waveform**	**Reversible Electroporation Area (mm^2^)**	**Electroporation Threshold (V/cm)**
2-2-2 μ s (n = 9)	13.8 ± 2.5	1037.0 ± 71.1
5-2-5 μ s (n = 8)	28.3 ± 4.0	781.9 ± 44.3
10-1-10 μ s (n = 9)	37.7 ± 10.0	708.1 ± 67.3
2-5-2 μ s (n = 9)	14.7 ± 2.1	1009.0 ± 54.7
5-5-5 μ s (n = 10)	27.2 ± 4.1	795.0 ± 43.4
**Waveform**	**H-FIRE Ablation Area (mm^2^)**	**Ablation Threshold (V/cm)**
2-2-2 μ s (n = 8)	10.6 ± 0.6	1244.8 ± 36.2
5-2-5 μ s (n = 8)	21.5 ± 1.5	904.2 ± 27.0
10-1-10 μ s (n = 9)	36.2 ± 1.7	725.1 ± 12.7
2-5-2 μ s (n = 8)	12.0 ± 1.2	1177.4 ± 53.2
5-5-5 μ s (n = 9)	22.4 ± 1.3	887.4 ± 21.7

Statistical analysis and figures are plotted in the supplementary materials (Figures S3 and S4).

**Table 3:** Electrical and thermal properties for numerical modeling.

Material	Parameter	Value	Units
Brain tissue	Density, ρ Specific heat, c_p_ Thermal conductivity, k Blood perfusion coefficient, ω Temperature coefficient, α	1045 3696 0.55 $1.75\times 10^{-3}$ 2	kg/m^3^ J/(kg·K) W/(m·K) 1/s %/°C
Insulation	Density, ρ Specific heat, c_p_ Thermal conductivity, k Electrical conductivity, σ	1190 1470 0.18 $1\times 10^{-10}$	kg/m^3^ J/(kg·K) W/(m·K) S/m
Stainless steel	Density, ρ Specific heat, c_p_ Thermal conductivity, k Electrical conductivity, σ	7850 475 44.5 $4.032\times 10^{6}$	kg/m^3^ J/(kg·K) W/(m·K) S/m

Material properties taken from either Lorenzo et al. [18] or the IT’IS database [46].

**Table 4:** Conductivity sigmoid parameters by characteristic frequency.

Waveform ( μ s)	Char. Frequency (kHz)	Conductivity σ _0_ (S/m)	E_delta_ (V/cm)	A (Unitless)
10-1-10	45.5	0.1267	725.1	1.302
5-5-5	50	0.1275	887.4	1.288
5-2-5	71.4	0.1306	904.2	1.234
2-5-2	71.4	0.1306	1177.4	1.234
2-2-2	125	0.1358	1244.8	1.148

Final conductivity ( σ _f_ at 10 MHz) = 0.2917 S/m; E_range_ = 350 V/cm.

## Data Availability

Not applicable.

## Supplementary Materials

The following are available online at https://www.mdpi.com/1424-8247/14/12/1333/s1, Figure S1: HF-PEF burst scheme nomenclature and representative voltage recordings, Figure S2: Reversible electroporation time-lapse study, Figure S3: Statistical analysis for healthy rodent astrocyte ablation and cell reversible electroporation in vitro, Figure S4: Statistical analysis for blood-brain barrier disruption in healthy rodent brain tissue in vivo, Figure S5: Visual depiction of H-FIRE cell ablation, reversible electroporation, BBB disruption, and nerve excitation effects resulting from HF-PEF treatment, Figure S6: Contributions of pulse width and interphase delay on BBB disruption, cell ablation, and nerve excitation evaluated across the monopolar, bipolar, and single needle grounding pad configurations, Figure S7: Direct, visual comparison of the cell ablation, blood-brain barrier disruption, and nerve excitation effects elicited during HF-PEF therapy at 2 kV applied, Figure S8: Electrical conductivity sigmoids for the 2-2-2 μ s, 2-5-2 μ s, 5-2-5 μ s, 5-5-5 μ s, and 10-1-10 μ s waveforms, Table S1: Summary of in vivo BBB disruption for PEF treatment in literature, Video S1: In vivo nerve excitation comparison of 2-5-2 μ s and 5-5-5 μ s HF-PEF treatment using a single-needle grounding pad configuration.
