# Supplementary material for: An Investigation for Large Volume, Focal Blood-Brain Barrier Disruption with High-Frequency Pulsed Electric Fields
Source: Pharmaceuticals (Basel). 2021 Dec 20;14(12):1333. doi: 10.3390/ph14121333 (PMC8715747; doi:10.3390/ph14121333)
Supplement: Supplementary file 1 [file pharmaceuticals-14-01333-s001.zip › SupplementalMaterial.pdf]

Supplementary Materials: An Investigation for Large Volume, Focal Blood-Brain Barrier Disruption with High-Frequency Pulsed Electric Fields

Melvin F. Lorenzo[1,†,*], Sabrina N. Campelo[1,†], Julio P. Arroyo[1], Kenneth N. Aycock[1], Jonathan Hinckley[2], Christopher B. Arena[1], John H. Rossmeisl Jr.[2], Rafael V. Davalos[1]

1. Collagen extraction

Briefly, extraction of collagen was performed as described previously [1,2] by having collagen I harvested from the rat tail tendons of Sprague Dawley rats and dissolved in 0.1 M hydrochloric acid overnight with continuous agitation. The suspension was centrifuged at 3000 × g for 45 minutes at 4°C and the supernatant was decanted. The final concentration was determined by dry-weight measurements and the collagen was sterilized by layering chloroform underneath overnight.

2. Quantification of Evans Blue Dye fluorescence

EBD fluorescence in brain tissue and blood samples were processed using a previously described dye extraction method [3,4]. Blood samples were centrifuged for 10 min at 10,000 × g at 4°C. The supernatants were aspirated and mixed (1:3 v/v) with 50% trichloroacetic acid (TCA; dissolved in 0.9% saline, Sigma; St. Louis, MO, USA). This solution was then centrifuged (10,000 × g for 10 min at 4°C). The resulting supernatant was collected, diluted with 50% TCA (1:300 v/v), and then again in 95% ethanol (1:3 v/v). The brain samples were homogenized in 50% TCA (1:3 w/v) using a steel-bead homogenizer (Beadblaster, Thomas Scientific, Swedesboro, NJ, USA), centrifuged (10,000 x g for 10 min at 4°C), and the supernatants collected and diluted with 95% ethanol (1:3 v/v) prior to spectrophotometric determination of EBD fluorescence. The final TCA extracted supernatants were loaded onto a 96-well plate in duplicate (30 µl/well), and EBD fluorescence of blood and brain tissues was determined using a spectrophotometer (620nm excitation/680nm emission; SpectraMax Plus, Molecular Devices, San Jose, CA, USA).

3. Supporting figures

Table S1 summarizes BBB disruption volumes and electric field thresholds from studies of Sharabi et al. [5], Hjouj et al. [6], and Garcia et al. [7].

Table S1. Summary of in vivo BBB disruption for PEF treatment in literature

Voltage (V)	On Time (μs)	Hz	Pulse #	BBB Vol (mm^3)	IRE Vol (mm^3)	Ratio	Ref
600	50	1	10	8.18*	1.00*	8.18	[5] BBBd: 500 V/cm IRE: 700 V/cm
			45	22.07*	10.31*	2.14	
			90	26.09*	2.95*	8.84	
			180	68.64*	11.99*	5.72	
			270	38.79*	10.77*	3.60	
			450	95.95*	29.65*	3.24	
			540	81.54*	24.43*	3.34	
250	50	4	90	24.50	4.17	5.88	[6] BBBd: 330 V/cm IRE: 500 V/cm
300				28.00	5.88	4.76	
350				35.20	13.38	2.63	
600				109.80	48.31	2.27	
650				162.20	92.45	1.75	
160	50	1	90	11.82	1.520**	1.76***	[7] BBBd: 400-600 V/cm IRE: – V/cm
240				8.97	3.140**	1.06***	
320				19.55	4.830**	1.73***	
400				32.00	4.510**	0.84***	

∗ measurements calculated from reported radii assuming spherical volumes
∗∗ indicates that only a cross sectional area was reported (units mm^2)
∗ ∗ ∗ indicates ratio was computed using cross sectional areas of IRE and BBBd reported

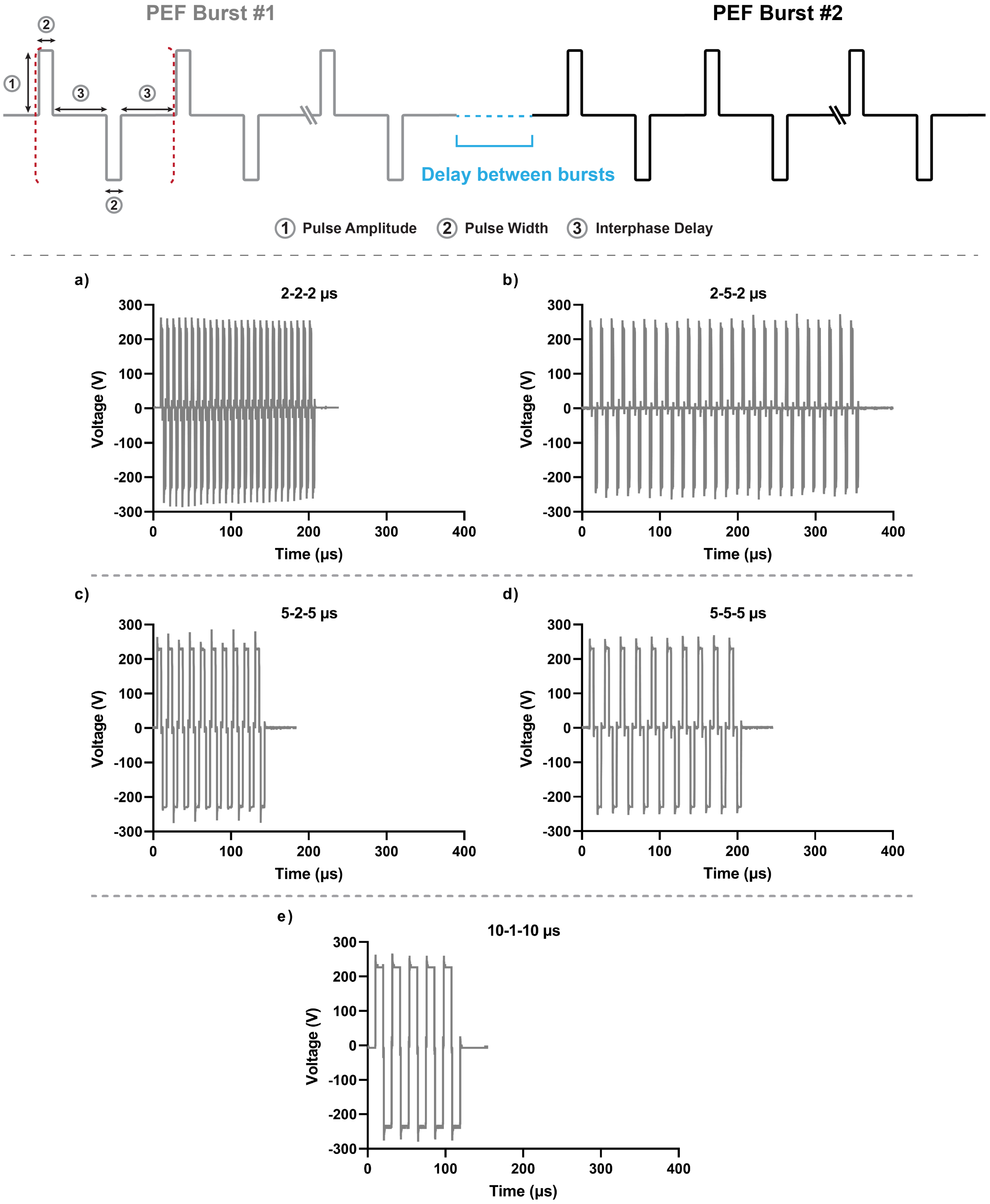

Figure S1. The HF-PEF burst scheme nomenclature is: positive phase – interphase delay – negative phase *μ*s. In this study, pulse width and interphase delay were varied to modulate the extent of cell death in relation to BBB disruption. The waveforms are (a) 2-2-2 *μ*s, (b) 2-5-2 *μ*s, (c) 5-2-5 *μ*s, (d) 5-5-5 *μ*s, and (e) 10-1-10 *μ*s burst schemes as recorded from *in vivo* treatments. HF-PEF bursts, each with an on-time of 100 *μ*s, were delivered once every second for a total treatment time of 200 seconds. To maintain a voltage-distance ratio of 600 V/cm *in vivo*, a potential 240 V was applied.

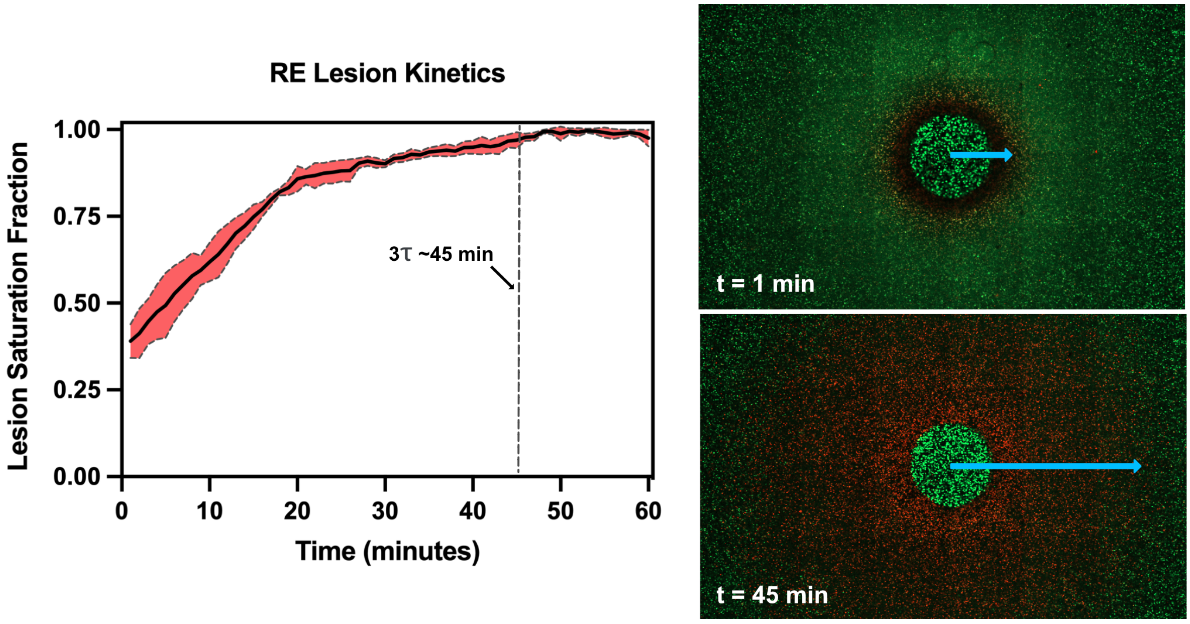

Figure S2. Time-lapse of reversible electroporation to characterize transient progression in the collagen hydrogel tissue mimic. One-minute after pulsing, the hydrogel was imaged once every 30 seconds and the relative size of the propidium iodide uptake region quantified. Sample size for the time-lapse study was n = 3.

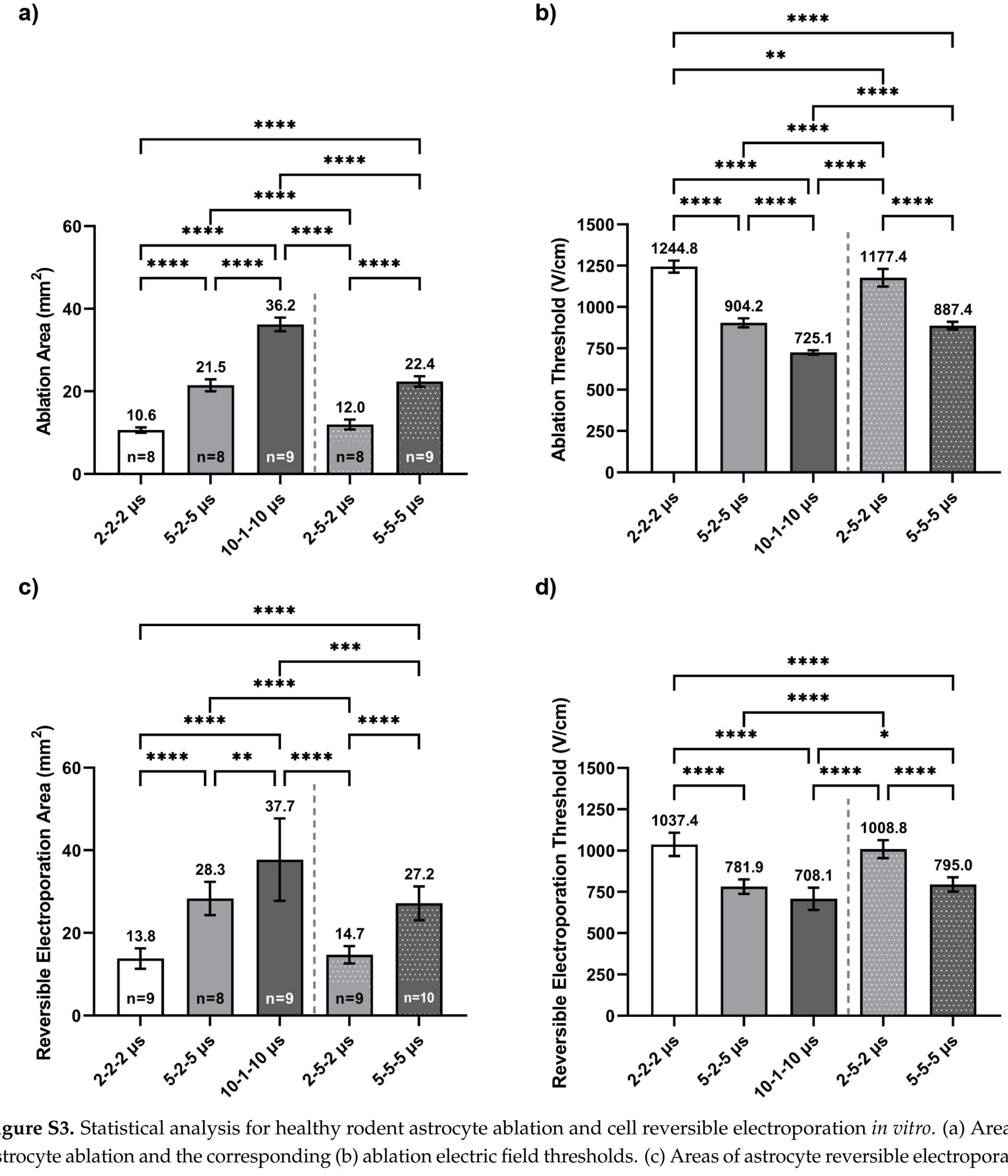

Figure S3. Statistical analysis for healthy rodent astrocyte ablation and cell reversible electroporation *in vitro*. (a) Areas of astrocyte ablation and the corresponding (b) ablation electric field thresholds. (c) Areas of astrocyte reversible electroporation and the corresponding (d) reversible electroporation electric field thresholds. * denotes a p-value $\leq$ 0.05, ** denotes a p-value $\leq$ 0.01, *** denotes a p-value $\leq$ 0.001, and **** denotes a p-value $\leq$ 0.0001.

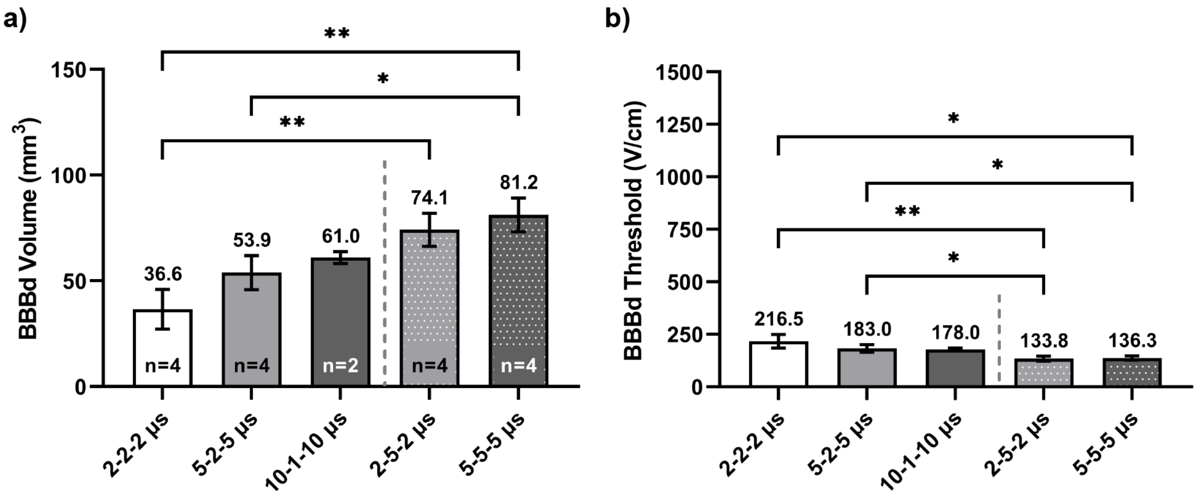

Figure S4. Statistical analysis for blood-brain barrier disruption in healthy rodent brain tissue *in vivo*. (a) Volumes of BBBd disruption measured from gross pathological tissue sections and the corresponding (b) BBBd electric field thresholds. * denotes a p-value $\leq$ 0.05, ** denotes a p-value $\leq$ 0.01.

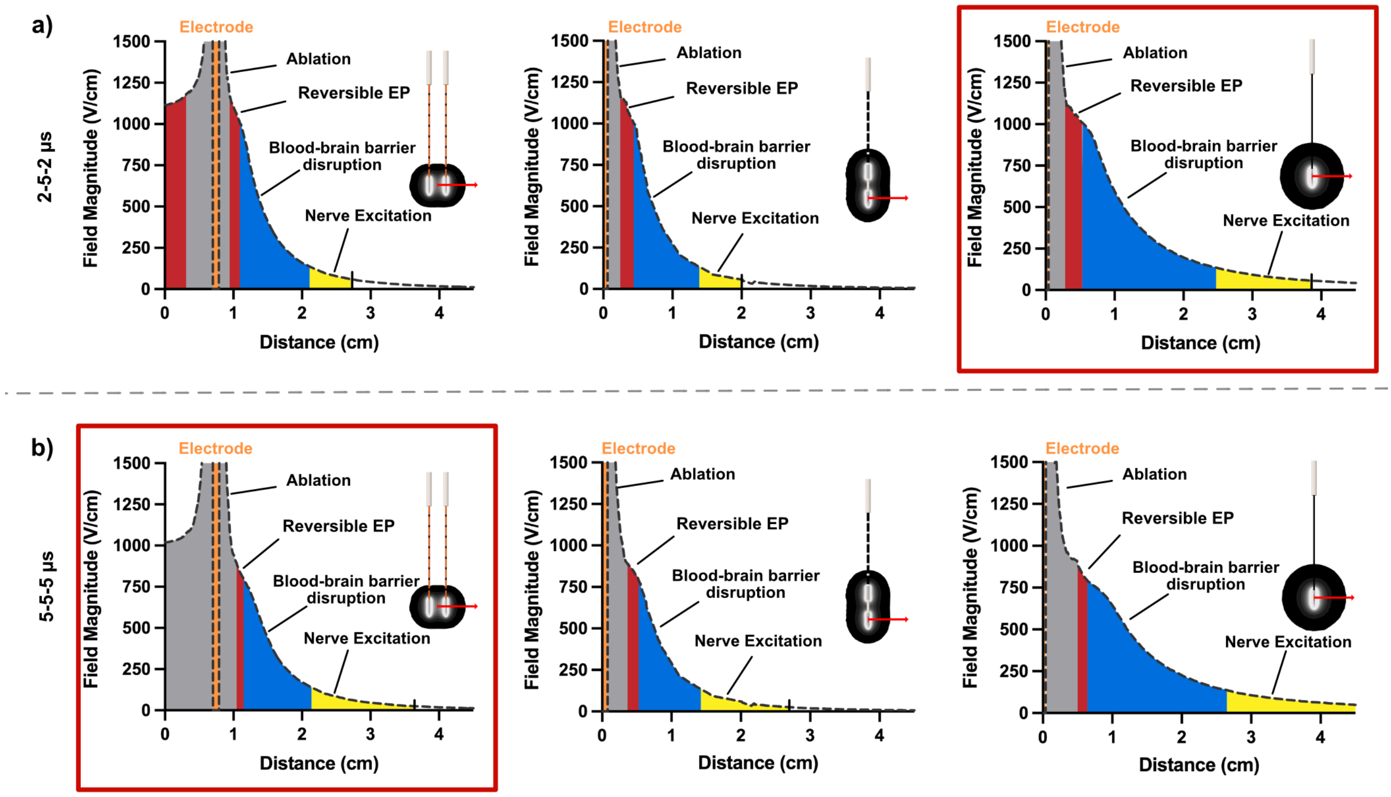

Figure S5. Visual depiction of H-FIRE cell ablation, reversible electroporation, BBB disruption, and nerve excitation effects resulting from HF-PEF treatment with the a) 2-5-2 μs burst scheme and the b) 5-5-5 μs burst scheme. This comparison is demonstrated across three distinct, clinically relevant electrode configurations. The single needle gounding pad configuration for the 2-5-2 μs waveform highlights a BBB disruption dominant protocol whereas the monopolar configuration for the 5-5-5 μs waveform results in larger volumes of ablation and reversible electroporation induction.

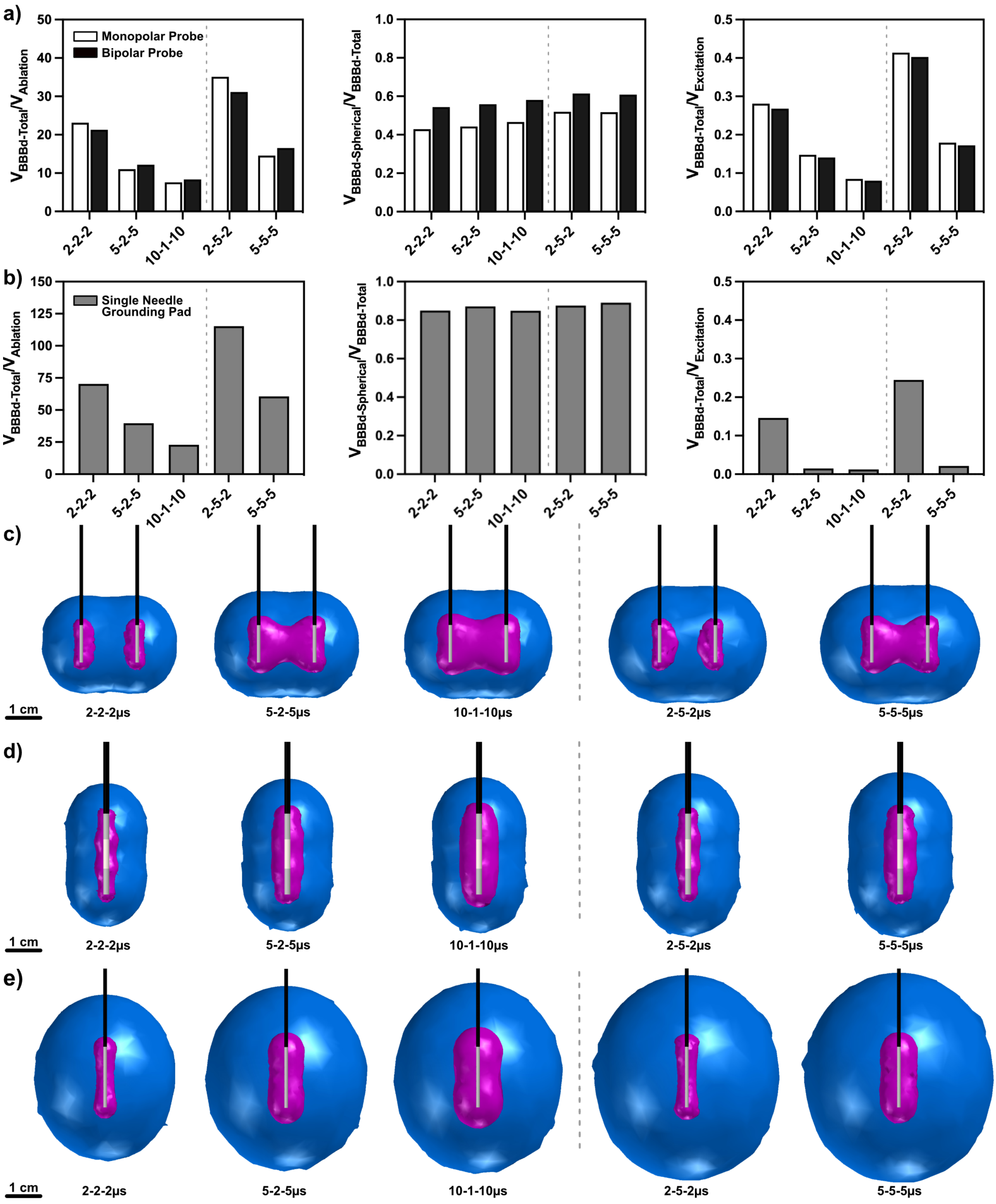

Figure S6. Contributions of pulse width and interphase delay on BBB disruption, cell ablation, and nerve excitation were evaluated across the (a,c) monopolar, (a,d) bipolar, and (b,e) single needle grounding pad configurations. (a,b) Components of Equation 1 were evaluated across all waveforms for each electrode configuration. Three-dimensional contours across waveforms and configurations (c-d) were generated to scale to make direct comparisons of ablation (magenta) and BBB disruption (blue) volumes. In general, the single needle grounding pad maximizes on the $V_{BBBd\text{-}Total}/V_{Ablation}$ ratio, whereas the monopolar probe capitalizes on high electric fields near and adjacent to the electrode for large ablation and reversible electroporation regions.

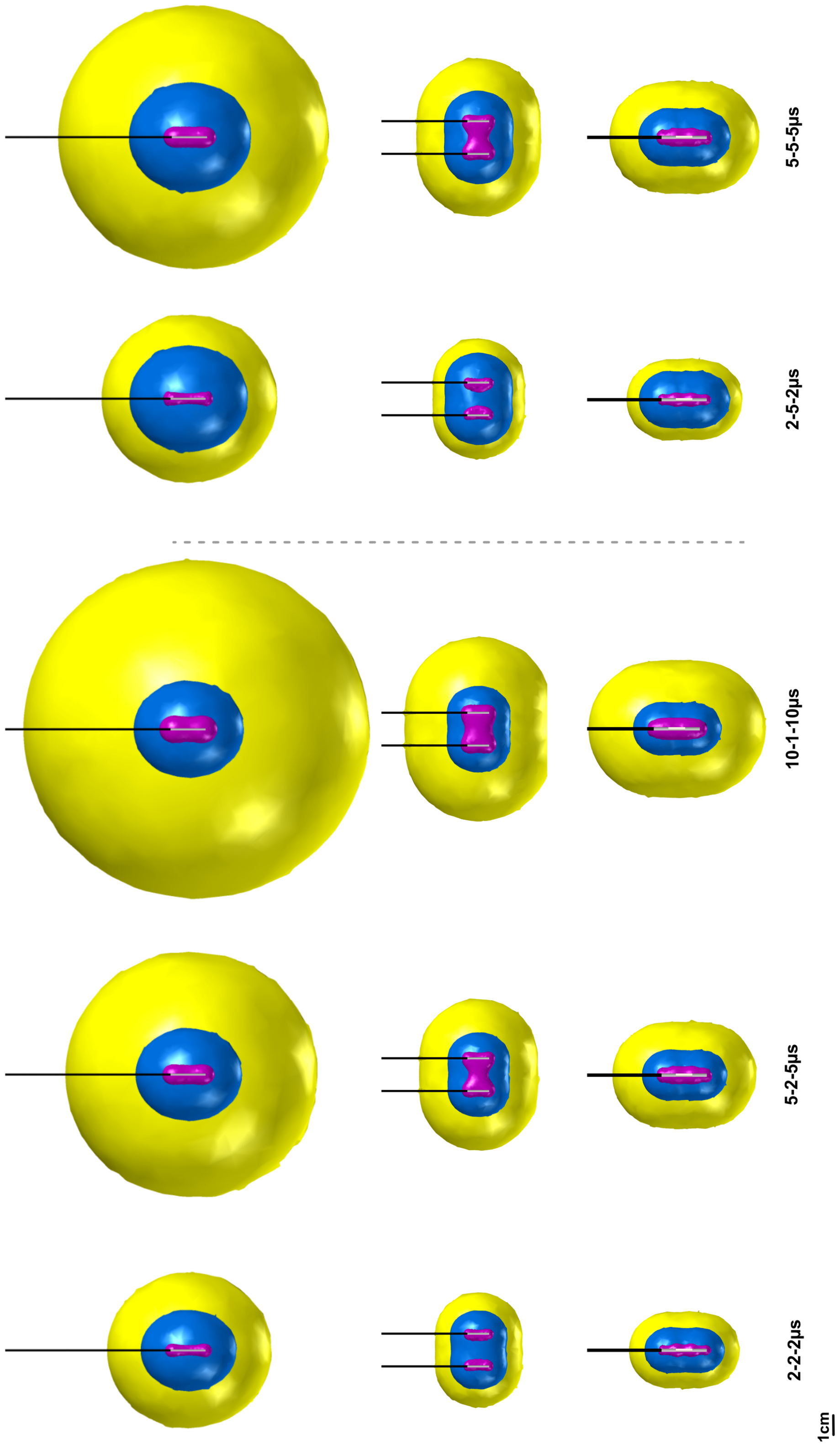

Figure S7. Direct, visual comparison of the cell ablation, blood-brain barrier disruption, and nerve excitation effects elicited during HF-PEF therapy at 2kV applied. These numerical simulation are specific to the 2-2-2 μs, 5-2-5 μs, 10-1-10 μs, 2-5-2 μs, and 5-5-5 μs waveforms and are simulated to include electroporation effects and coupled Joule heating effects. Yellow represents volumes of tissue susceptible to nerve excitation, blue represents volumes of tissue in which BBB is disrupted, and magenta the expected ablation volume.

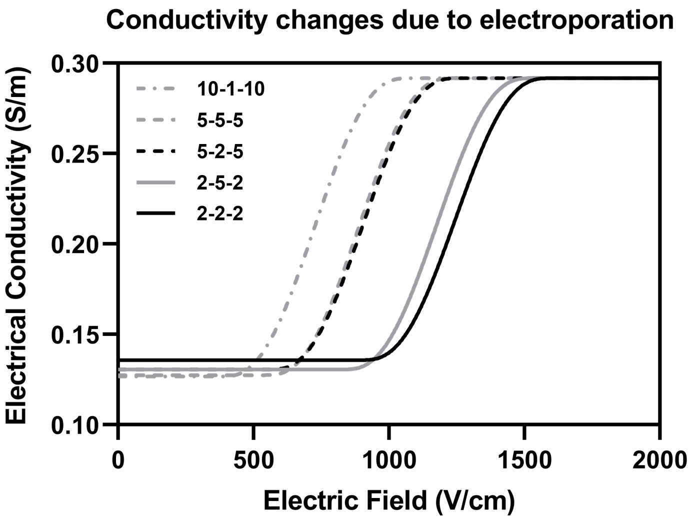

Figure S8. Electrical conductivity sigmoids for the 2-2-2 μs, 2-5-2 μs, 5-2-5 μs, 5-5-5 μs, and 10-1-10 μs waveforms were constructed. The non-electroporated conductivity σ_0 is the conductivity of grey matter brain tissue at the characteristic frequency of each waveform and the electroporated conductivity σ_f is the conductivity of grey matter at 10 MHz. E_{delta} is equal to the lethal EFT for each waveform, and E_{range} is set to 350 V/cm for all waveforms.

References

1. Arena, C.B.; Szot, C.S.; Garcia, P.A.; Rylander, M.N.; Davalos, R.V. A three-dimensional in vitro tumor platform for modeling therapeutic irreversible electroporation. *Biophysical journal* **2012**, *103*, 2033–2042.
2. Wasson, E.M.; Alinezhadbalalami, N.; Brock, R.M.; Allen, I.C.; Verbridge, S.S.; Davalos, R.V. Understanding the role of calcium-mediated cell death in high-frequency irreversible electroporation. *Bioelectrochemistry* **2020**, *131*, 107369.
3. Uyama, O.; Okamura, N.; Yanase, M.; Narita, M.; Kawabata, K.; Sugita, M. Quantitative evaluation of vascular permeability in the gerbil brain after transient ischemia using Evans blue fluorescence. *Journal of Cerebral Blood Flow & Metabolism* **1988**, *8*, 282–284.
4. Lorenzo, M.F.; Thomas, S.C.; Kani, Y.; Hinckley, J.; Lee, M.; Adler, J.; Verbridge, S.S.; Hsu, F.C.; Robertson, J.L.; Davalos, R.V.; others. Temporal characterization of blood–brain barrier disruption with high-frequency electroporation. *Cancers* **2019**, *11*, 1850.
5. Sharabi, S.; Kos, B.; Last, D.; Guez, D.; Daniels, D.; Harnof, S.; Mardor, Y.; Miklavcic, D. A statistical model describing combined irreversible electroporation and electroporation-induced blood-brain barrier disruption. *Radiology and oncology* **2016**, *50*, 28.
6. Hjouj, M.; Last, D.; Guez, D.; Daniels, D.; Sharabi, S.; Lavee, J.; Rubinsky, B.; Mardor, Y. MRI study on reversible and irreversible electroporation induced blood brain barrier disruption. *PloS one* **2012**.
7. Garcia, P.A.; Rossmeisl Jr, J.H.; Robertson, J.L.; Olson, J.D.; Johnson, A.J.; Ellis, T.L.; Davalos, R.V. 7.0-T magnetic resonance imaging characterization of acute blood-brain-barrier disruption achieved with intracranial irreversible electroporation. *PloS one* **2012**, *7*, e50482.
