# Supplementary material for: An Investigation for Large Volume, Focal Blood-Brain Barrier Disruption with High-Frequency Pulsed Electric Fields
Source: Pharmaceuticals (Basel). 2021 Dec 20;14(12):1333. doi: 10.3390/ph14121333 (PMC8715747; doi:10.3390/ph14121333)
Supplement: Supplementary file 1 [file pharmaceuticals-14-01333-s001.zip › Latex Source for PDF.zip!/Definitions/pharmaceuticals-logo-eps-converted-to.pdf]

pharmaceuticals
